# Changes in Weight, Waist Circumference or Both With Incident Heart Failure in Chinese Middle‐Aged and Older Adults

**DOI:** 10.1002/jcsm.70059

**Published:** 2025-09-18

**Authors:** Yu Yin, Rui Tang, Xi Wang, Mengyi Zheng, Jingli Qu, Shuohua Chen, Shouling Wu, Yu Yuan

**Affiliations:** ^1^ Department of Occupational and Environment Health. Key Laboratory of Environment and Health, Ministry of Education and State Key Laboratory of Environmental Health (Incubating) School of Public Health, Tongji Medical College, Huazhong University of Science and Technology Wuhan China; ^2^ Cardiovascular Center Beijing Tongren Hospital, Capital Medical University Beijing China; ^3^ Department of Cardiology Kailuan General Hospital Tangshan China

**Keywords:** cardiovascular disease, middle‐aged and elderly population, prospective study, waist change, weight change

## Abstract

**Background:**

Previous studies have acknowledged that higher body weight and waist circumference were associated with an increased risk of heart failure. Notably, both body weight and waist circumference can change over time. However, no previous study has investigated the association between combined changes in weight and waist circumference in middle‐aged and older adults and incident heart failure.

**Methods:**

This prospective study included 45 620 middle‐aged and older Chinese adults (aged 45–104 years). These participants were free of critical diseases at baseline, including coronary heart disease, stroke, heart failure, atrial fibrillation and cancer. Weight change from 2006–2007 to 2012–2013 was categorized into five groups: excessive weight loss (change < −10%, *N* = 3943), lesser weight loss (−10% ≤ change < −5%, *N* = 5890), stable weight (±5%, *N* = 23 208), lesser weight gain (5% < change ≤ 10%, *N* = 7153) and excessive weight gain (> 10%, *N* = 5426). Waist circumference change was categorized into five groups: excessive waist circumference loss (change < −10%, *N* = 8236), lesser waist circumference loss (−10% ≤ change < −5%, *N* = 6215), stable waist circumference (±5%, *N* = 16 953), lesser waist circumference gain (5% < change ≤ 10%, *N* = 6642) and excessive waist circumference gain (> 10%, *N* = 7574). Combined changes in weight and waist circumference were divided into 25 groups, i.e., cross‐classified combinations derived from the five categories of weight change and five categories of waist circumference change. Incident heart failure cases that occurred from 2012–2013 to December 31, 2022 were recorded. Cox proportional hazards regression models were used to estimate the associations of weight change, waist circumference change or both with heart failure. Multivariate models were stratified by age at risk (in 5‐year intervals) and sex, and were adjusted for variables including height, smoking, drinking, educational attainment, occupation, dietary pattern, physical activity, hypertension, fasting blood glucose and total serum cholesterol. In the analysis of weight change, we additionally adjusted for weight at baseline and waist circumference change. Conversely, for the analysis of waist circumference change, adjustments were made for baseline waist circumference and weight change. When examining combined weight and waist circumference changes, adjustments were made for both baseline weight and waist circumference. Additionally, we employed restricted cubic spline analyses to examine the nonlinear associations between changes in weight or waist circumference and heart failure.

**Results:**

We identified 1036 heart failure cases during follow‐up. The median (interquartile range, IQR) of follow‐up time was 9.66 (9.40, 9.96) years. The incidence rate of heart failure was 2.47 cases per 1000 person‐years. The median (IQR) age of our participants was 52.1 (46.8, 57.7) years. The proportion of men was 77.9%. The mean (standard deviation) of weight and waist circumference at baseline (baseline, 2006–2007) was 70.1 (10.4) kg and 87.2 (9.0) cm, respectively. Compared with those who kept stable weight, participants in the excessive weight gain group had a higher risk (HR [hazard ratio], 1.27; 95% CI [confidence interval]: 1.03–1.57). Compared with those who kept stable waist circumference, participants in the excessive waist circumference gain group had a higher risk (HR, 1.28; 95% CI: 1.05–1.56), while those in the excessive waist circumference loss group had a lower risk of heart failure (HR, 0.76; 95% CI: 0.64–0.92). Compared with participants with stable weight and waist circumference, those who lost excessive weight and kept stable waist circumference (HR, 1.53; 95% CI: 1.10–2.14), those who lost lesser weight and gained excessive waist circumference (HR, 2.19; 95% CI: 1.38–3.46), and those who gained excessive weight and excessive waist circumference (HR, 1.48; 95% CI: 1.03–2.14) had a higher risk of heart failure. The restricted cubic spline illustrated a U‐shaped relation between weight change and incident heart failure (*P* overall = 0.027, *P* for non‐linear relation = 0.007), whereas a positive linear relation was observed for waist circumference change with incident heart failure (*p* overall < 0.001, *p* for non‐linear relation = 0.675).

**Conclusions:**

Excessive weight gain and waist circumference gain were associated with 27% and 28% higher risk of heart failure, while excessive waist circumference loss was associated with a 24% lower risk of heart failure.

## 1 Introduction

Heart failure (HF) has been defined as a global pandemic, with the increase of the global number of HF cases from 27.2 million in 1990 to 55.5 million in 2021 [1]. Prior studies have acknowledged that obesity (measured by body mass index [BMI] and waist circumference [WC]) is a major risk factor for HF, which suggested that both higher BMI and higher WC were associated with increased risk of HF [2]. Nevertheless, these studies only included the baseline values rather than the long‐term changes of anthropometric indicators [2], while both body weight and WC may change over time [3]. Evaluating the changes in weight and WC may more accurately reflect long‐term adiposity status of individuals and provide additional information on fat redistribution [4]. Several epidemiological studies have explored the associations of changes in weight with the risk of incident HF [5, 6, 7], which suggested that the top and bottom 10% of weight changes [5], the weight change in the highest and third quartiles compared to that in the lowest quartile [6] and weight gain of 10 kg or more [7] were associated with a higher risk of HF. Nonetheless, the weight changes in these studies occurred during puberty (8–20 years) [6] or from young to middle adulthood [7], not in middle and old age. Additionally, these studies were limited by special populations (patients with Type 2 diabetes [T2D] and either established cardiovascular disease [CVD] or at high CVD risk [5] and the males [6]). Moreover, two trials conducted in the United States and Canada involving patients with T2D have explored the association between changes in WC and the incidence of HF [8, 9]. These studies found that a loss of WC was associated with a lower risk of HF [8], while a gain in WC was linked to a higher risk of HF [9]. Compared with a single absolute change, relative change can reduce the bias caused by baseline differences. However, the above studies [5, 6, 7, 8, 9] only included the absolute changes in weight and WC; few studies [5, 6, 7, 8, 9] have examined the relationships between weight or WC relative change during middle and old age and the risk of incident HF.

Although the individuals may experience concurrent increases or decreases of both body weight and WC in most circumstances [10], body weight and WC may also change in different directions. Specifically, sarcopenia has been characterized as an age‐related decline in skeletal muscle mass, which may result in weight loss in the elderly [11]. Meanwhile, older adults experience an increase in WC due to age‐related adipose tissue accumulation and redistribution [12]. For instance, in the Australian Diabetes, Obesity and Lifestyle study, adults who were 25–34 years of age gained an average of 6.7 kg weight and 6.6 cm WC during a 12‐year follow‐up, whereas those aged ≥ 75 years lost an average of 4.5 kg weight and gained 0.8 cm WC [3]. Despite the fact that middle‐aged and older adults may experience simultaneous changes in weight and WC [13], there is a lack of studies investigating the association of the combined changes in weight and WC with the development of HF, especially in middle‐aged and older adults.

To fill in the knowledge gap, we aimed to examine the associations of changes in weight or WC, especially the combined changes of them with incident HF in middle‐aged and older Chinese adults.

## 2 Methods

### 2.1 Study Population

Participants were from the Kailuan cohort, which is an ongoing prospective cohort study conducted in Tangshan, China. From July 2006 to October 2007 (baseline), the Kailuan cohort enrolled 101 510 participants from the Kailuan Company, and all the participants underwent questionnaire interviews, clinical examinations and laboratory tests at enrollment. They were resurveyed every 2 years. There were 33 840 individuals lost to follow up at visit (2012–2013). A total of 67 670 participants were surveyed, both at baseline (2006–2007) and during visit 3 (2012–2013). Participants who met the following criteria were excluded: (1) pregnant during the baseline or visit 3 (*N* = 112); (2) diagnosed with coronary heart disease, stroke, HF, atrial fibrillation or cancer at baseline or visit 3 (*N* = 3274); (3) with missing date of baseline or visit 3 (*N* = 1); (4) with missing weight or WC data at baseline or visit 3 (*N* = 5761); (5) with baseline age < 40 years (*N* = 12 155); (6) with abnormal value of weight or WC at baseline or visit 3 (*N* = 734); and (7) with BMI < 15 or > 50 kg/m^2^ at baseline or visit 3 (*N* = 13). Finally, we included 45 620 participants in the analyses (Figure S1). The study was conducted following the guidelines of the Declaration of Helsinki and was approved by the Kailuan General Hospital Ethics Committee. Our study follows the Strengthening the Reporting of Observational Studies in Epidemiology (STROBE) reporting guideline for cohort studies. The Kailuan Study was registered with the Chinese Clinical Trial Registry (ChiCTR‐TNC‐11001489). All the participants agreed to participate in the study and provided written informed consent.

### 2.2 Assessment of Changes in Weight and WC

In the Kailuan cohort, standing height and body weight were measured using standard instruments and protocols with participants barefoot and wearing light indoor clothing [14]. WC was measured using a soft non‐stretchable tape at the midpoint between the lowest rib margin and iliac crest.

The changes in weight and WC were calculated by subtracting the values obtained at baseline (2006–2007) from those obtained at the follow‐up visit (visit 3, 2012–2013) and then dividing the results by the values obtained at baseline. Changes in weight were categorized into five groups as follows: excessive weight loss (change < −10%), lesser weight loss (−10% ≤ change < −5%), stable weight (±5%), lesser weight gain (5% < change ≤ 10%) and excessive weight gain (> 10%). Changes in WC were categorized into five groups as follows: excessive WC loss (change < −10%), lesser WC loss (−10% ≤ change < −5%), stable WC (±5%), lesser WC gain (5% < change ≤ 10%) and excessive WC gain (> 10%). The stable weight and stable WC groups were defined as the reference groups for weight change and WC change, respectively. Combined changes in weight and WC were categorized into 25 groups, reflecting the combination of five levels of weight change (excessive weight loss, lesser weight loss, stable weight, lesser weight gain and excessive weight gain) and five levels of WC change (excessive WC loss, lesser WC loss, stable WC, lesser WC gain and excessive WC gain). For the 25 groups, the stable weight and stable WC group was set as the reference group.

### 2.3 Assessment of Outcomes

In the Kailuan cohort, each participant was followed from visit 3 (2012–2013) to the incidence of HF, death, or the end of follow‐up (December 31, 2022), whichever came first. HF cases were identified through a rigorous process. Two experienced cardiologists (both professors with Doctor of Medicine degrees), who were blinded to the current study protocol, independently reviewed the medical records to confirm the first occurrence of HF. In cases where there was disagreement between the two cardiologists, a third cardiologist (also a professor with a Doctor of Medicine degree) was consulted to provide the final adjudication. The method of HF diagnosis has been described in detail previously [15]. Specifically, we followed the International Classification of Disease 10th revision codes I50.x to identify HF cases [15]. By reviewing the medical records of the participants, it was clear that (1) the presence of symptoms indicative of HF, such as dyspnoea, fatigue and fluid retention; additionally, the cardiac function at discharge was classified based on the New York Heart Association cardiac function Grade II, III or IV [16] or Killip II, III or IV [17]; (2) left ventricular ejection fraction ≤ 50% measured using 2‐dimensional and Doppler echocardiography according to the modified Simpson's method [18]; and (3) left ventricular ejection fraction > 50% but with elevated plasma N‐terminal pro‐brain natriuretic peptide (NT‐proBNP) levels (≥ 125 ng/L) [19]. The diagnosis of HF must meet the criteria (1) and any of (2) and (3) [15].

### 2.4 Assessment of Covariates

BMI was calculated as weight (kg) divided by the square of height (m^2^). Smoking status was categorized as never, current and former smokers. Alcohol intake status was categorized as never, current and former drinkers. Educational attainment was divided into three categories: primary school or below, middle school and high school or beyond. Occupations were classified based on participants' work settings—specifically, whether they worked in the mine, and the nature of their labour, distinguishing between manual and mental tasks. Those engaged in manual labour within the mine were categorized as coal miners. Participants who undertook mental labour within the mine or manual labour outside the mine were classified as other blue‐collar workers. Finally, individuals performing mental labour outside the mine were designated as white‐collar workers [20]. Dietary patterns were evaluated based on the questions in the questionnaire regarding the frequency of tea drinking (≥ 4 times/week, 1–3 times/week, 1–3 times/month, < 1 time/month and never), the frequency of consuming fatty food (< 1 time/week, 1–3 times/week and > 3 times/week), and the daily salt intake (< 6 g/day, 6–12 g/day and > 12 g/day) [20]. Dietary scores were applied to assess the quality of dietary patterns. The frequency categories for tea consumption were assigned scores of 100, 75, 50, 25 and 0, respectively. Similarly, the frequency categories for fatty food intake were assigned scores of 100, 50 and 0. The categories for daily salt consumption were also assigned scores of 100, 50 and 0. By summing the scores from these three questions, the total score was standardized and reassigned a value ranging from 0 to 100. Dietary pattern was classified into three groups: favourable pattern (80–100), intermediate pattern (50–79) and unfavourable pattern (0–49) [20]. Physical activity (PA) was self‐reported by participants. Participants were categorized into three groups based on their reported frequency and duration of PA: no (< 1 time/week), occasional (1–2 times/week) or regular (≥ 3 times/week and ≥ 30 min/time) PA [21]. Blood pressure was measured twice at a 5‐min interval on the day of physical examination using a mercury sphygmomanometer on the left upper arm with each participant in the seated position. The average of two readings was used for the analysis. If the difference between the two readings was more than 5 mmHg, a third measurement was taken, and the average of the three readings was used for the analysis [22]. Hypertension was defined as systolic blood pressure ≥ 140 mmHg, diastolic blood pressure ≥ 90 mmHg, the use of antihypertensive medication or self‐reported hypertension [22]. The concentrations of fasting blood glucose and total cholesterol were measured using the Hitachi 747 auto‐analyser (Hitachi, Tokyo, Japan) on the day of the blood draw.

### 2.5 Statistical Analysis

Continuous variables with a normal distribution were described using means (standard deviations, SD); non‐normally distributed continuous variables were described using medians (interquartile ranges, IQR) and categorical variables were described using frequencies (percentages). The One‐way ANOVA and Kruskal–Wallis tests were used to compare differences in normally distributed and non‐normally distributed continuous variables across groups defined by changes in weight and WC, respectively. The Chi‐square test was employed to compare differences in categorical variables among these groups. Person‐years for each participant were calculated from the date of the follow‐up visit (visit 3, 2012–2013) until the date of HF, death, or the end of follow‐up (December 31, 2022), whichever came first. The incidence rate of HF was calculated by dividing the number of incident cases by the total follow‐up duration (person‐years).

The Kaplan–Meier method was used to analyse time‐to‐event data and group differences were compared using the log‐rank test. Cox proportional hazards regression models were used to estimate the association of weight change, WC change or both with incident HF. The proportional hazard assumptions were evaluated by visualization of Schoenfeld residuals, and the assumption was valid. Multivariate models were stratified by age at risk (5‐year interval) [23] and sex, and were adjusted for height, smoking status (never, former or current smoker), alcohol intake status (never, former or current drinker), educational attainment (primary school or below, middle school, high school or beyond), occupations (coal miners, other blue‐collar workers or white‐collar workers), dietary pattern (favourable, intermediate and unfavourable patterns), PA (no, occasional or regular), hypertension status (yes or no), fasting blood glucose and total serum cholesterol levels. Age‐at‐risk is a time‐varying covariate whereby, as an individual gets older during the follow‐up, they may contribute to more than one ‘age‐at‐risk’ group [24]. For the analysis of weight change, we additionally adjusted for weight at cohort recruitment and WC change. For the analysis of WC change, we additionally adjusted for WC at cohort recruitment and weight change. For the analysis of weight and WC changes, we additionally adjusted for weight and WC at cohort recruitment. To examine the nonlinear associations of changes in weight or WC with HF, we further utilized restricted cubic splines (RCS) analyses. The analyses were performed using three knots positioned at the 5th, 50th and 95th percentiles of the distribution of weight and WC changes. The reference values (HR = 1) were set at the point where weight and WC changes were equal to zero. The confounders included in this analysis were consistent with those used in Cox model 3 for weight and WC change analyses. Nonlinearity was examined using likelihood ratio tests.

In the stratified analyses, we further examined the associations of changes in weight, WC or both with incident HF among prespecified baseline subgroups based on age (< 65 years and ≥ 65 years), sex (male and female), BMI at recruitment (< 24 kg/m^2^ and ≥ 24 kg/m^2^), WC (< 85 cm for male or < 80 cm for female and ≥ 85 cm for male or ≥ 80 cm for female), PA level (low or high) [25] and dietary pattern (favourable or unfavourable). PA level was divided into low PA (no PA) and high PA (occasional and regular PA). Dietary pattern was divided into favourable pattern (favourable pattern and intermediate pattern) and unfavourable pattern (unfavourable pattern).

Several sensitive analyses were conducted to test the robustness of the results. First, we restricted the sample to those who have never smoked given the impact of smoking on the HF risk. Second, we excluded HF cases that occurred within the first 1 year after the follow‐up visit 3 (2012–2013) to minimize potential reverse causality. Third, Fine and Grey subdistribution models were also performed to validate the analysis in case of the presence of competing risks of death.

Missing covariates were recoded with missing indicators for categorical variables or median for continuous variables. Analysis was performed with the SAS program (version 9.4) and R software (version 4.3.3). Two‐sided *p* < 0.05 was considered statistically significant.

## 3 Results

### 3.1 Study Participants

Our study included 45 620 participants, with a total of 1036 incident HF cases identified during the follow‐up period (2012–2022). The median follow‐up time was 9.66 years (IQR: 9.40, 9.96), and the incidence rate of HF was 2.47 cases per 1000 person‐years. The median age of participants was 52.1 years (IQR: 46.8, 57.7) and 77.9% (35 520/45620) were men. At baseline (2006–2007), the mean weight was 70.1 kg (SD: 10.4), and the mean WC was 87.2 cm (SD: 9.0). From baseline to the follow‐up visit (2012–2013), the mean change in weight was 0.3 kg (SD: 6.7), and the mean change in WC was −0.6 cm (SD: 9.9), over a median duration of 6.33 years (IQR: 6.00, 6.61). Compared to the stable weight group, participants in the weight loss group were older, had a higher proportion of males, heavier baseline weight, larger baseline WC, and a higher proportion of PA, non‐smoking, non‐drinking, unfavourable dietary patterns, lower education levels and hypertension (Table 1). Compared to the stable WC group, participants in the WC loss group were older, had a lower proportion of males and hypertension, heavier baseline weight, larger baseline WC, and a higher proportion of PA, non‐smoking, non‐drinking, unfavourable dietary patterns and education levels (Table 2). The basic characteristics of participants grouped by weight and WC changes were shown in Tables 1 and 2.

**TABLE 1:** Basic characteristics of the participants by weight change (*n* = 45 620).

	Overall	Weight change (%)					*p* value
		Loss		Stable	Gain		
Characteristics^a^		(Change < −10)	(−10 ≤ Change < −5)	(−5 ≤ Change ≤ 5)	(5 < Change ≤ 10)	(Change > 10)	
No. (%) of participants	45 620 (100.0%)	3943 (8.6%)	5890 (12.9%)	23 208 (50.9%)	7153 (15.7%)	5426 (11.9%)	
Incidence rate, per 1000 person‐years	2.5 (2.3, 2.6)	3.3 (2.8, 4.0)	2.7 (2.3, 3.2)	2.3 (2.1, 2.6)	2.3 (2.0, 2.7)	2.4 (2.0, 2.9)	
Age at cohort recruitment, years	52.1 (46.8, 57.7)	53.2 (47.7, 59.2)	52.7 (47.5, 58.3)	51.9 (46.8, 57.5%)	51.6 (46.5, 57.0)	52.1 (46.4, 58.5)	< 0.001
Men	35 520 (77.9%)	3135 (79.5%)	4706 (79.9%)	18 141 (78.2%)	5461 (76.3%)	4077 (75.1%)	< 0.001
Weight at cohort recruitment, kg	70.1 (10.4)	77.6 (9.9)	73.7 (9.6)	70.7 (9.9)	67.1 (9.5)	62.5 (8.3)	< 0.001
BMI at cohort recruitment^b^, kg/m^2^	25.1 (3.2)	27.5 (3.3)	26.2 (3.0)	25.3 (3.0)	24.11 (2.9)	22.7 (2.7)	< 0.001
Height at cohort recruitment, cm	167.1 (6.9)	168.0 (6.9)	167.7 (6.9)	167.2 (6.9)	166.7 (6.8)	165.8 (6.8)	< 0.001
Waist circumference at cohort recruitment, cm	87.2 (9.0)	90.4 (8.9)	89.1 (8.6)	87.5 (8.8)	85.7 (9.0)	83.4 (8.9)	< 0.001
Physical activity^c^							< 0.001
No physical activity	10 855 (23.8%)	865 (21.9%)	1203 (20.4%)	5620 (24.2%)	1841 (25.7%)	1326 (26.4%)	
Occasional physical activity	28 671 (62.8%)	2535 (64.3%)	3835 (65.1%)	14 465 (62.3%)	4419 (61.8%)	3417 (63.0%)	
Regular physical activity	5917 (13.0%)	534 (13.5%)	823 (14.0%)	3035 (13.1%)	866 (12.1%)	659 (12.1%)	
Smoking status^c^							< 0.001
Never smoker	31 329 (68.7%)	2834 (71.9%)	4158 (70.6%)	15 773 (68.0%)	4854 (67.9%)	3710 (68.4%)	
Former smoker	1585 (3.5%)	112 (2.8%)	188 (3.2%)	828 (3.6%)	250 (3.5%)	207 (3.8%)	
Current smoker	12 516 (27.4%)	986 (25.0%)	1514 (25.7%)	6514 (28.1%)	2018 (28.2%)	1484 (27.3%)	
Alcohol intake^c^							< 0.001
Never drinker	33 250 (72.9%)	3047 (77.3%)	4434 (75.3%)	16 615 (71.6%)	5158 (72.1%)	3999 (73.6%)	
Former drinker	127 (0.3%)	11 (0.3%)	17 (0.3%)	72 (0.3%)	20 (0.3%)	7 (0.1%)	
Current drinker	12 060 (26.4%)	876 (22.2%)	1412 (24.0%)	6426 (27.7%)	1948 (27.2%)	1398 (25.8%)	
Occupation^c^							< 0.001
Coal miners	8222 (18.0%)	692 (17.6%)	1065 (18.1%)	4197 (18.1%)	1308 (18.3%)	960 (17.7%)	
Other blue‐collar workers	30 838 (67.6%)	2691 (68.3%)	4014 (68.1%)	15 550 (67.0%)	4786 (66.9%)	3797 (70.0%)	
White‐collar workers	5421 (11.9%)	440 (11.2%)	667 (11.3%)	2906 (12.5%)	887 (12.4%)	521 (9.6%)	
Dietary pattern^c^							< 0.001
Unfavourable	30 765 (67.5%)	2779 (70.5%)	4046 (68.8%)	15 408 (66.4%)	4764 (66.6%)	3768 (69.5%)	
Intermediate	13 710 (30.1%)	1075 (27.3%)	1691 (28.7%)	7184 (31.0%)	2220 (31.1%)	1540 (28.4%)	
Favourable	1110 (2.4%)	88 (2.2%)	146 (2.5%)	598 (2.6%)	165 (2.3%)	113 (2.1%)	
Educational attainment^c^							< 0.001
Primary school or below	3620 (7.9%)	362 (9.2%)	504 (8.6%)	1729 (7.5%)	494 (6.9%)	531 (9.8%)	
Middle school	35 873 (78.6%)	3073 (77.9%)	4617 (78.4%)	18 212 (78.5%)	5714 (79.9%)	4257 (78.5%)	
High school or beyond	6123 (13.4%)	508 (12.9%)	768 (13.0%)	3266 (14.1%)	944 (13.2%)	637 (11.7%)	
Hypertension	21 228 (46.7%)	1892 (48.1%)	2812 (47.9%)	10 830 (46.9%)	3325 (46.7%)	2369 (43.9%)	< 0.001
FBG, mmol/L	5.4 (4.9, 6.1)	5.4 (4.9, 6.1)	5.5 (4.9, 6.3)	5.4 (5.0, 6.1)	5.4 (4.9, 6.0)	5.3 (4.9, 5.9)	< 0.001
TC, mmol/L	5.2 (1.6)	5.1 (1.2)	5.1 (1.6)	5.2 (1.7)	5.2 (1.0)	5.2 (1.8)	< 0.001

^a^ Continuous variables were presented as mean (SD) or median (IQR) and categorical variables were presented as *n* (%).

^b^ BMI was weight in kilograms divided by height in meters squared.

^c^ Data were incomplete for these variables. In the Kailuan study, 0.4% (*n* = 177), 0.4% (*n* = 190), 0.4% (*n* = 183), 0.1% (*n* = 35), 0% (*n* = 4) and 2.5% (*n* = 1139) of participants had missing data for physical activity, smoking status, alcohol intake, dietary pattern, educational attainment and occupation, respectively. The other variables included in the analyses did not have missing data.

Abbreviations: BMI, body mass index; FBG, fasting blood glucose; IQR, interquartile range; SD, standard deviation; TC, total cholesterol.

**TABLE 2:** Basic characteristics of the participants by waist circumference change (*n* = 45 620).

	Overall	Waist circumference change (%)					*p* value
		Loss		Stable	Gain		
Characteristics^a^		(Change < −10)	(−10 ≤ Change < −5)	(−5 ≤ Change ≤ 5)	(5 < Change ≤ 10)	(Change > 10)	
No. (%) of participants	45 620 (100.0%)	8236 (18.1%)	6215 (13.6%)	16 953 (37.2%)	6642 (14.6%)	7574 (16.6%)	
Incidence rate, per 1000 person‐years	2.5 (2.3, 2.6)	2.6 (2.3, 3.0)	2.9 (2.5, 3.4)	2.5 (2.2, 2.7)	2.1 (1.8, 2.5)	2.3 (2.0, 2.7)	
Age at cohort recruitment, years	52.1 (46.8, 57.7)	53.6 (48.3, 60.3)	52.9 (47.9, 58.9)	52.0 (46.8, 57.5)	51.1 (45.4, 56.4)	51.1 (45.7, 56.5)	< 0.001
Men	35 520 (77.9%)	5997 (72.8%)	4857 (78.1%)	13 484 (79.5%)	5244 (79.0%)	5938 (78.4%)	< 0.001
Weight at cohort recruitment, kg	70.1 (10.4)	69.7 (10.5)	71.1 (10.2)	70.8 (10.2)	70.0 (10.4)	68.4 (10.6)	< 0.001
BMI at cohort recruitment^b^, kg/m^2^	25.1 (3.2)	25.3 (3.4)	25.5 (3.2)	25.2 (3.1)	24.9 (3.1)	24.3 (3.3)	< 0.001
Height at cohort recruitment, cm	167.1 (6.9)	165.9 (7.2)	167.0 (6.8)	167.4 (6.8)	167.5 (6.8)	167.5 (6.9)	< 0.001
Waist circumference at cohort recruitment, cm	87.2 (9.0)	93.1 (8.7)	90.2 (8.1)	87.5 (7.9)	84.2 (7.7)	80.1 (7.7)	< 0.001
Physical activity ^c^							< 0.001
No physical activity	10 855 (23.8%)	1127 (13.7%)	1305 (21.0%)	4651 (27.4%)	1920 (28.9%)	1852 (24.5%)	
Occasional physical activity	28 671 (62.8%)	6549 (79.5%)	4214 (67.8%)	9748 (57.5%)	3601 (54.2%)	4559 (60.2%)	
Regular physical activity	5917 (13.0%)	535 (6.5%)	683 (11.0%)	2486 (14.7%)	1087 (16.4%)	1126 (14.9%)	
Smoking status^c^							< 0.001
Never smoker	31 329 (68.7%)	6574 (79.8%)	4477 (72.0%)	11 228 (66.2%)	4184 (63.0%)	4866 (64.2%)	
Former smoker	1585 (3.5%)	177 (2.2%)	212 (3.4%)	632 (3.7%)	273 (4.1%)	291 (3.8%)	
Current smoker	12 516 (27.4%)	1458 (17.7%)	1512 (24.3%)	5019 (29.6%)	2151 (32.4%)	2376 (31.4%)	
Alcohol intake^c^							< 0.001
Never drinker	33 250 (72.9%)	6858 (83.3%)	4681 (75.3%)	11 947 (70.5%)	4462 (67.2%)	5302 (70.0%)	
Former drinker	127 (0.3%)	13 (0.2%)	23 (0.4%)	59 (0.4%)	17 (0.3%)	15 (0.2%)	
Current drinker	12 060 (26.4%)	1339 (16.3%)	1498 (24.1%)	4875 (28.8%)	2128 (32.0%)	2220 (29.3%)	
Occupation^c^							< 0.001
Coal miners	8222 (18.0%)	1003 (12.2%)	978 (15.7%)	3294 (19.4%)	1416 (21.3%)	1531 (20.2%)	
Other blue‐collar workers	30 838 (67.6%)	6212 (75.4%)	4353 (70.0%)	11 067 (65.3%)	4237 (63.8%)	4969 (65.6%)	
White‐collar workers	5421 (11.9%)	735 (8.9%)	688 (11.1%)	2242 (13.2%)	871 (13.1%)	885 (11.7%)	
Dietary pattern^c^							< 0.001
Unfavourable	30 765 (67.5%)	6720 (81.7%)	4370 (70.3%)	10 642 (62.8%)	4057 (61.1%)	4976 (65.8%)	
Intermediate	13 710 (30.1%)	1407 (17.1%)	1734 (27.9%)	5809 (34.3%)	2372 (35.7%)	2388 (31.6%)	
Favourable	1110 (2.4%)	101 (1.2%)	109 (1.8%)	488 (2.9%)	209 (3.2%)	203 (2.7%)	
Educational attainment^c^							< 0.001
Primary school or below	3620 (7.9%)	558 (6.8%)	499 (8.0%)	1335 (7.9%)	591 (8.9%)	637 (8.4%)	
Middle school	35 873 (78.6%)	6853 (83.2%)	4979 (80.1%)	13 131 (77.5%)	5016 (75.5%)	5894 (77.8%)	
High school or beyond	6123 (13.4%)	824 (10.0%)	737 (11.9%)	2485 (14.7%)	1035 (15.6%)	1042 (13.8%)	
Hypertension	21 228 (46.7%)	3809 (46.5%)	2983 (48.2%)	8066 (47.8%)	3022 (45.7%)	3348 (44.4%)	< 0.001
FBG, mmol/L	5.4 (4.9, 6.1)	5.4 (4.9, 6.1)	5.4 (4.9, 6.1)	5.4 (4.9, 6.1)	5.4 (5.0, 6.0)	5.4 (5.0, 6.0)	0.654
TC, mmol/L	5.2 (1.6)	5.1 (1.5)	5.1 (1.6)	5.2 (1.6)	5.2 (1.6)	5.2 (1.6)	< 0.001

^a^ Continuous variables were presented as mean (SD) or median (IQR) and categorical variables were presented as *n* (%).

^b^ BMI was weight in kilograms divided by height in meters squared.

^c^ Data were incomplete for these variables. In the Kailuan study, 0.4% (*n* = 177), 0.4% (*n* = 190), 0.4% (*n* = 183), 0.1% (*n* = 35), 0% (*n* = 4) and 2.5% (*n* = 1139) of participants had missing data for physical activity, smoking status, alcohol intake, dietary pattern, educational attainment and occupation, respectively. The other variables included in the analyses did not have missing data.

Abbreviations: BMI, body mass index; FBG, fasting blood glucose; IQR, interquartile range; SD, standard deviation; TC, total cholesterol.

### 3.2 Changes in Weight, Waist Circumference or Both With Incident Heart Failure

**FIGURE 1:**
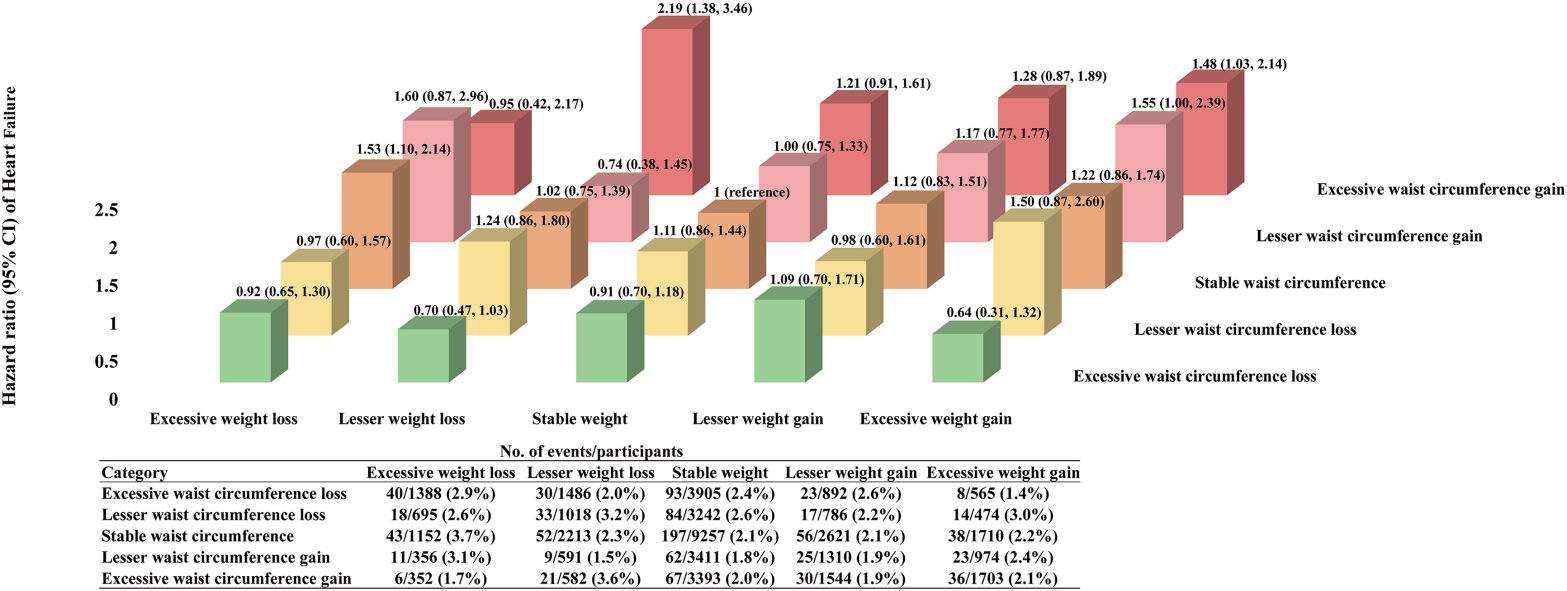
Adjusted hazard ratios for heart failure based on the combined changes in weight and waist circumference. The multivariable‐adjusted model included the combined categories of weight and waist circumference changes, weight, height and waist circumference at cohort recruitment, smoking status, alcohol intake status, dietary pattern, educational attainment, physical activity, occupation, hypertension, fasting blood glucose, total serum cholesterol level; and stratified by age at risk (5‐year interval) and sex. Weight changes were categorized into five groups: Excessive weight loss (lost > 10%), lesser weight loss (10% ≤ lost < 5%), stable weight (change within 5%), lesser weight gain (5% < gained ≤ 10%) and excessive weight gain (gained > 10%). Waist circumference changes were categorized into five groups: Excessive waist circumference loss (lost > 10%), lesser waist circumference loss (10% ≤ lost < 5%), stable waist circumference (change within 5%), lesser waist circumference gain (5% < gained ≤ 10%) and excessive waist circumference gain (gained > 10%).

**FIGURE 2:**
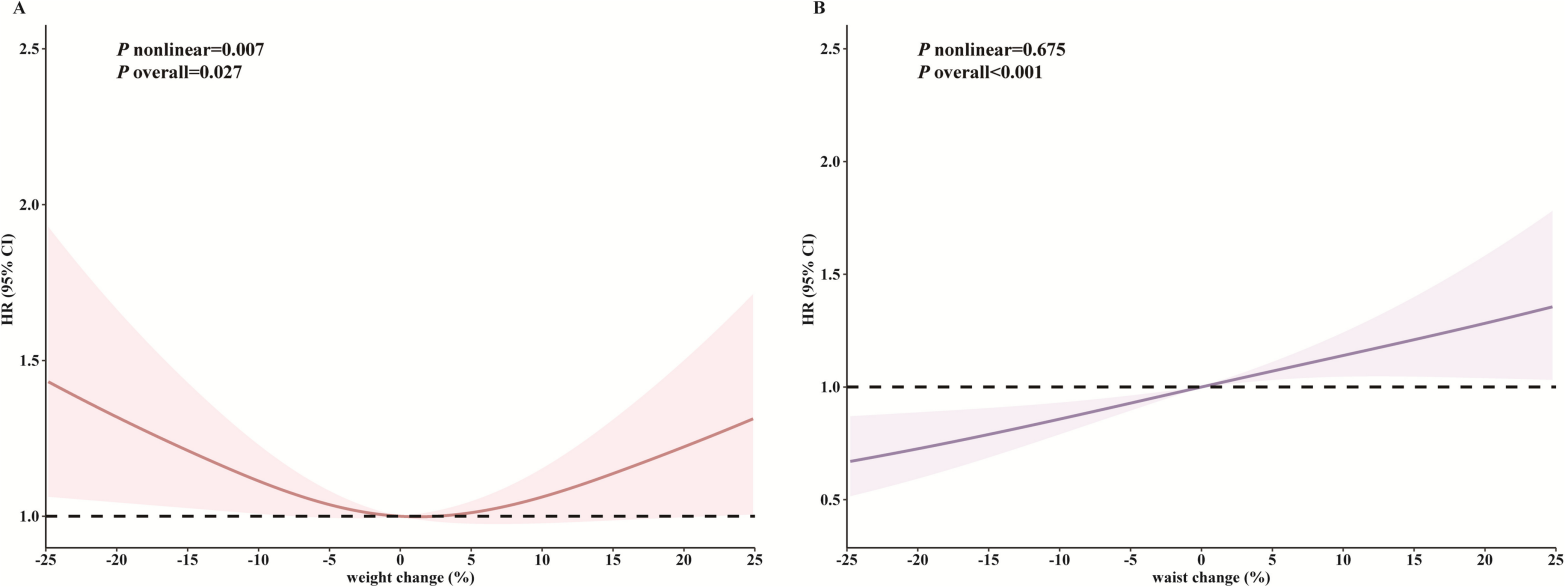
The restricted cubic spline for the association between weight and waist circumference change categories and heart failure. The curve represents adjusted HRs based on restricted cubic splines with knots at the 5th, 50th and 95th percentiles of the distribution of weight and waist circumference percent change (solid lines are HRs, dashed lines indicate 95% CIs), the reference values (HR = 1) were set at where weight and waist circumference changes were equal to zero. The multivariable‐adjusted models were stratified by age at risk (5‐year interval), sex and adjusted for height, weight change, waist circumference change, smoking status, alcohol intake status, dietary pattern, educational attainment, physical activity, occupation, hypertension, fasting blood glucose and total serum cholesterol level. For the analysis of weight change, we additionally adjusted for weight at cohort recruitment. For the analysis of waist circumference change, we adjusted for waist circumference at cohort recruitment.

We identified 1036 incident HF cases during 419 565 person‐years follow‐up. The person‐years of follow‐up associated with the endpoints of HF, death, and the end of follow‐up were 4272, 24 361 and 390 932, respectively. There were statistically significant differences observed among the survival curves of the weight change groups, as well as among the survival curves of the WC change groups (log‐rank *p* < 0.001, Figure S2). Compared to the stable weight group, the other four weight change groups had a higher cumulative incidence of HF (Figure S2A). In contrast, the groups experiencing waist circumference gain demonstrated a higher cumulative incidence of HF compared to the stable WC group, while the WC loss groups exhibited a lower cumulative incidence of HF (Figure S2B). After adjustments for covariates, we found a significant relation between excessive weight gain and higher risk of incident HF compared to stable weight. The HR (hazard ratio, 95% confidence interval, CI) of the excessive weight gain group was 1.27 (95% CI: 1.03–1.57) in the fully adjusted model (Table 3). Compared with those in the stable WC group, the participants in the excessive WC gain group had a 28% higher risk of HF (adjusted HR, 1.28; 95% CI: 1.05–1.56), while the participants in the excessive WC loss group had a 24% lower risk of HF (adjusted HR, 0.76; 95% CI: 0.64–0.92) (Table 4). For the combined changes of weight and WC, compared with the stable weight and WC group, those who maintained stable WC and lost excessive weight had a higher risk of HF, with adjusted HR (1.53; 95% CI: 1.10–2.14). Those who gained excessive WC and lost lesser weight had a higher risk of HF, with adjusted HR (2.19; 95% CI: 1.38–3.46). Those who gained excessive WC and gained excessive weight had a higher risk of HF, with adjusted HR (1.48; 95% CI: 1.03–2.14) (Figure 1). The restricted cubic spline (RCS) showed an approximate U‐shape for the relation between weight change and incident HF (*p* overall = 0.027, *p* for non‐linear relation = 0.007). Both weight loss and weight gain were related to higher risks of HF (Figure 2A). The RCS analysis showed a significantly positive linear dose–response association between WC change and the risk of HF (*p* overall < 0.001, *p* for non‐linear relation = 0.675). WC loss was related to lower risk of HF, while WC gain was related to the higher risk of HF (Figure 2B).

**TABLE 3:** Associations between weight change categories and heart failure.

	Weight change (%)				
	Loss		Stable	Gain	
Variable	(Change < −10)	(−10 ≤ Change < −5)	(−5 ≤ Change ≤ 5)	(5 < Change ≤ 10)	(Change > 10)
No. of events/total	118/3943	145/5890	503/23208	151/7153	119/5426
Incidence rate, per 1000 person‐years	3.33 (2.78, 3.99)	2.70 (2.30, 3.18)	2.34 (2.14, 2.55)	2.28 (1.95, 2.68)	2.42 (2.02, 2.90)
HR (95% CI)					
Model 1	1.05 (0.85, 1.30)	1.01 (0.84 1.21)	1 (reference)	1.12 (0.93, 1.34)	**1.28 (1.04, 1.58)**
Model 2	1.03 (0.83, 1.27)	1.00 (0.83, 1.21)	1 (reference)	1.12 (0.93, 1.35)	**1.28 (1.04, 1.58)**
Model 3	1.10 (0.89, 1.36)	1.00 (0.83, 1.21)	1 (reference)	1.11 (0.93, 1.34)	**1.27 (1.03, 1.57)**

Model 1: The multivariable models were adjusted for height and weight at cohort recruitment, waist circumference change (continuous variables) and stratified by age at risk (5‐year interval) and sex.

Model 2: The multivariable models were adjusted for height and weight at cohort recruitment, waist circumference change (continuous variables), smoking status, alcohol intake status, dietary pattern, educational attainment, physical activity and stratified by age at risk (5‐year interval) and sex.

Model 3: The multivariable models were adjusted for height and weight at cohort recruitment, waist circumference change (continuous variables), smoking status, alcohol intake status, dietary pattern, educational attainment, physical activity, occupation, hypertension, fasting blood glucose, total serum cholesterol level and stratified by age at risk (5‐year interval) and sex.

Abbreviations: CI, confidence interval; HR, hazard ratio.

**TABLE 4:** Associations between waist circumference change categories and heart failure.

	Waist circumference change (%)				
	Loss		Stable	Gain	
Variable	(Change < −10)	(−10 ≤ Change < −5)	(−5 ≤ Change ≤ 5)	(5 < Change ≤ 10)	(Change > 10)
No. of events/total	194/8236	166/6215	386/16953	130/6642	160/7574
Incidence rate, per 1000 person‐years	2.61 (2.26, 3.00)	2.94 (2.53, 3.42)	2.46 (2.23, 2.72)	2.10 (1.77, 2.49)	2.29 (1.96, 2.67)
HR (95% CI)					
Model 1	**0.73 (0.61, 0.88)**	1.00 (0.83, 1.20)	1 (reference)	1.06 (0.86, 1.29)	**1.33 (1.10, 1.62)**
Model 2	**0.72 (0.60, 0.87)**	1.00 (0.83, 1.20)	1 (reference)	1.05 (0.86, 1.29)	**1.33 (1.10, 1.62)**
Model 3	**0.76 (0.64, 0.92)**	1.02 (0.85, 1.23)	1 (reference)	1.04 (0.85, 1.27)	**1.28 (1.05, 1.56)**

Model 1: The multivariable models were adjusted for height and waist circumference at cohort recruitment, weight change (continuous variables) and stratified by age at risk (5‐year interval) and sex.

Model 2: The multivariable models were adjusted for height and waist circumference at cohort recruitment, weight change (continuous variables), smoking status, alcohol intake status, dietary pattern, educational attainment, physical activity and stratified by age at risk (5‐year interval) and sex.

Model 3: The multivariable models were adjusted for height and waist circumference at cohort recruitment, weight change (continuous variables), smoking status, alcohol intake status, dietary pattern, educational attainment, physical activity, occupation, hypertension, fasting blood glucose, total serum cholesterol level and stratified by age at risk (5‐year interval) and sex.

Abbreviations: CI, confidence interval; HR, hazard ratio.

### 3.3 Stratified Analyses

There were no clear differences in associations of weight change, WC change or both with the risk of incident HF according to strata of age, sex, BMI, WC, PA and dietary pattern (Figures S3–S10). The relatively small sample size in specific strata may lead to insufficient statistical power, thereby limiting the ability of those variables to modify the effects of changes in weight, WC or both on the risk of HF (Figures S3–S10).

### 3.4 Sensitivity Analyses

In the sensitivity analyses, there was no substantial change in the association of weight change, WC change or both with HF among participants who have never smoked (*n* = 31 329) or in the analyses excluding HF cases that occurred within the first 1 year (*n* = 45 498) (Tables S1–S4, Figures S11–S12). Furthermore, the subdistribution HRs obtained from the Fine‐Grey model were still consistent with the main results (Tables S5 and S6, Figure S13). Thus, we considered competing risks of death not to be an issue in the Cox model.

## 4 Discussion

We examined the relationship between changes in weight and WC and the risk of incident HF in a large prospective cohort of middle‐aged and older Chinese adults. Key findings include a U‐shaped association between weight change and HF risk, and a positive linear association between WC change and HF risk. Additionally, individuals who experienced excessive weight loss with stable WC, those who lost lesser weight and gained excessive WC, or those who gained excessive weight and excessive WC had a higher risk of HF compared to those with stable measurements. These results underscore the importance of monitoring weight and WC changes for HF prevention in middle‐aged and older adults. Taken together, prioritizing the monitoring of weight and WC changes in middle‐aged and older adults would be important for the precise prevention of HF.

Many previous studies have explored the association between weight change and the risk of HF [5, 6, 7]. However, these studies have generated disparate findings for the association of weight change with incident HF [5, 6, 7]. A study from the international multicenter CANVAS and CREDENCE trials demonstrated that extremes of weight loss were associated with a higher risk of HF in the CANVAS trial, whereas extremes of weight gain were linked to an increased risk of HF in the CREDENCE trial [5]. The BMI Epidemiology Study conducted in Sweden found that subjects in the highest and third quartiles of BMI change during puberty had a higher risk of HF compared to those in the lowest quartile, revealing a J‐shaped nonlinear association between BMI change and HF [6]. Additionally, another study from the United States indicated that weight gain of ≥ 10 kg was associated with a higher risk of HF, while the relationship between weight loss and HF risk was not statistically significant [7]. In our current study, a U‐shaped association between weight change and risk of HF was found. These inconsistent findings could be partly explained by different population characteristics (race, lifestyle, genetic susceptibility and pre‐existing disease) [5, 6, 7], self‐reported values of weight [7] and weight change in different age groups [5, 6, 7]. Meanwhile, there is scarce evidence regarding the association between changes in WC and incident HF in the general middle‐aged and older adults. A randomized clinical trial involving adults with T2D who were overweight or obese, with an average age of approximately 60 years, demonstrated that a reduction in WC was associated with a lower risk of HF [8]. Another randomized controlled trial conducted in the United States and Canada, which included patients with T2D, CVD or those at high risk for CVD, also with an average age of around 60 years, suggested that an increase in WC was more closely linked to the development of HF. Furthermore, a J‐shaped relationship was observed between changes in WC and the incidence of HF [9]. The existing studies [8, 9], which were conducted among participants with chronic diseases or at high CVD risk, may have limited generalizability due to their specific participant selection. In our study, we found a positive linear dose–response relation between WC change and incident risk of HF in general middle‐aged and older Chinese adults. WC gain is a strong indicator of abdominal fat accumulation, particularly visceral adipose tissue (VAT) [26]. The accumulation of VAT is associated with a higher content of free fatty acid (FFA) [27], which can impair cardiac structure and function through lipotoxicity [28]. Additionally, VAT adipocytes secrete pro‐inflammatory cytokines, including leptin, tumour necrosis factor‐alpha and interleukin‐6 [29]. These cytokines contribute to chronic inflammation, which may lead to the development of cardiac systolic dysfunction and myocardial fibrosis [29]. Given these mechanisms, it is plausible that WC gain, as a proxy for VAT accumulation, is related to a higher risk of HF independent of overall body weight change.

Weight is a measure of overall obesity, while WC is a surrogate measure of upper body or abdominal obesity [26]. The elder adults experience both weight and WC changes along with the ageing process [30]. A study from the Charleston Heart Study cohort showed that weight increased in adults who were 37–46 years of age, while weight decreased in55–74 years old adults [30]. However, WC of the adults in both age groups increased [30]. Therefore, when evaluating body size of the older people, both weight and WC measures should be taken into account and measuring weight as well as WC may further stratify individuals in the risk prediction of CVD. A previous Swedish prospective cohort study has investigated the combined associations of BMI and WC with HF, which showed that subjects with high BMI and high WC had higher risks of HF hospitalization compared with those with low or normal BMI and WC [31]. However, BMI and WC in this study were measured only once at baseline, which limited the ability to capture temporal changes in adiposity and their potential influence on HF risk. Body weight and WC would change over time [3, 30]; therefore, evaluating the changes in weight and WC may serve as a more appropriate measurement of long‐term adiposity status for the middle‐aged and older adults.

Our study found that compared to individuals maintaining stable weight and WC, higher risks of HF were observed in three groups, i.e., those with excessive weight loss but stable WC, those with lesser weight loss but excessive WC gain and those with excessive weight and WC gain. Weight loss in older adults is closely associated with sarcopenia [32]. Sarcopenia can contribute to the development and progression of HF through multiple pathophysiological pathways. First, sarcopenia is linked to chronic inflammation and oxidative stress, which result in endothelial dysfunction—a key factor in HF [33, 34]. Second, sarcopenia may contribute to the development of insulin resistance (IR), a known risk factor for incident HF [35, 36]. These may partially explain why individuals with weight loss but stable WC have a higher risk of HF compared to those maintaining stable weight and WC. While the present study did not measure body composition directly, future research should consider these factors to better understand the relationship between changes in weight and WC and the onset of sarcopenia and HF. In older adults, the phenotype of weight loss and WC gain may reflect sarcopenic obesity, which was defined as the co‐existence of sarcopenia and obesity [37]. As mentioned above, on the one hand, sarcopenia could increase the risk of HF by promoting inflammation, oxidative stress and IR mechanisms [33, 34, 35, 36], on the other hand, WC gain may lead to a higher risk of HF through many mechanisms, such as the accumulation of FFA or the release of more inflammatory factors [27, 28, 29]. A study from the UK Biobank showed that among participants who died from heart diseases (including heart failure), some had adverse muscle composition, characterized by low muscle volume coupled with high muscle fat infiltration [38]. This finding indirectly supports our finding that weight loss and waist circumference gain increase the risk of heart failure, as these changes may reflect an underlying muscle‐fat metabolic imbalance. The above mechanisms could offer a plausible interpretation for a higher risk of HF among individuals with weight loss and WC gain compared to those with stable weight and WC. Moreover, individuals with weight gain and WC gain should also be given attention, as their combined general obesity and abdominal adiposity may impose a higher risk of HF compared to those maintaining stable weight and WC, likely through pathways involving FFA accumulation [28] and release of pro‐inflammatory cytokines, including leptin, tumour necrosis factor‐alpha and interleukin‐6 [29]. Additionally, body fatness may be associated with altered left ventricular remodelling through increased haemodynamic load, neurohormonal activation and increased oxidative stress, which is a risk factor for HF [39].

The study found that the associations between changes in weight, WC or both and incident HF showed no significant differences across different dietary patterns. The non‐significant differences might be attributed to the relatively small sample sizes in subgroups after stratification. Importantly, these findings do not negate the critical role of dietary patterns in HF prevention. A favourable dietary pattern may still reduce HF risk through other pathways, such as modulating lipid profiles, blood pressure and blood glucose levels [40]. The dietary pattern stratification in our study was based on baseline dietary data and failed to dynamically track the impact of dietary pattern changes on study outcomes. Future research incorporating repeatedly measured dietary data could further explore whether the relationships between body size changes and HF differ in subgroups with dietary pattern modifications.

## 5 Study Advantages and Limitations

To the best of our knowledge, this is the first study to investigate the association of the combined changes in weight and WC with the risk of incident HF in a large prospective cohort study. Additionally, to further reduce the possibility of reverse causality, we performed analyses excluding HF cases that occurred within the first 1 year after the follow‐up visit (visit 3, 2012–2013) and generated similar findings. Several potential limitations should be noted. First, the generalizability of the study was limited, given the population was Chinese middle‐aged and older adults. The findings in this study need to be validated among other ethnic groups. Second, we only explored the association of the changes in weight and WC with HF of any cause and did not differentiate the specific types of HF. Third, we did not perform human body composition analysis and therefore could not distinguish between muscle mass and fat mass. However, changes in weight and WC may influence the risk of incident HF through overlapping effects of VAT accumulation, reduction in muscle mass or a systemic increase in total fat mass. These factors could collectively contribute to the observed associations. Therefore, more prospective studies evaluating the associations between changes in body composition and risk of HF are warranted in the future. Nonetheless, this study provides important evidence to inform public health interventions aimed at reducing HF risk through long‐term weight and WC management.

## 6 Conclusions

Our findings identified a U‐shaped association between weight change and incident HF, alongside a positive linear relationship between WC change and HF risk among Chinese middle‐aged and older adults. Notably, individuals with discordant weight and WC trajectories, specifically weight loss with stable WC, weight loss with WC gain or concurrent weight and WC gain, showed higher HF risks compared to those maintaining stable weight and WC. Our study underscores the importance of monitoring changes in both weight and WC when assessing the risk of incident HF in middle‐aged and older adults. These combined changes may provide valuable insights into HF risk. Further research is needed to elucidate the underlying mechanisms linking weight and WC changes to HF occurrence.

## Ethics Statement

The study was conducted with the guidelines of the Declaration of Helsinki and approved by the Kailuan General Hospital Ethics Committee. All participants agreed to participate in the study and provided written informed consent. The authors certify that they comply with the ethical guidelines for authorship and publishing in the Journal of Cachexia, Sarcopenia and Muscle [41].

## Conflicts of Interest

The authors declare no conflicts of interest.

## Supporting information


**Figure S1:** Flow chart of participant recruitment from the Kailuan study.
**Figure S2:** Cumulative incidence of heart failure by weight change and waist circumference change.
**Figure S3:** Associations between weight change categories and heart failure, stratified by age, sex, bmi, waist circumference, physical activity and dietary pattern.
**Figure S4:** Associations between waist circumference change categories and heart failure, stratified by age, sex, bmi, waist circumference, physical activity and dietary pattern.
**Figure S5:** Adjusted hazard ratios for heart failure stratified by age based on the combined changes in weight and waist circumference.
**Figure S6:** Adjusted hazard ratios for heart failure stratified by sex based on the combined changes in weight and waist circumference.
**Figure S7:** Adjusted hazard ratios for heart failure stratified by BMI based on the combined changes in weight and waist circumference.
**Figure S8:** Adjusted hazard ratios for heart failure stratified by waist circumference based on the combined changes in weight and waist circumference.
**Figure S9:** Adjusted hazard ratios for heart failure stratified by physical activity level based on the combined changes in weight and waist circumference.
**Figure S10:** Adjusted hazard ratios for heart failure stratified by dietary pattern based on the combined changes in weight and waist circumference.
**Figure S11:** Adjusted hazard ratios for heart failure among never smokers based on the combined changes in weight and waist circumference (*n* = 31329).
**Figure S12:** Adjusted hazard ratios for heart failure based on the combined changes in weight and waist circumference (excluding 122 heart failure cases within one year, *n* = 45498).
**Figure S13:** Adjusted hazard ratios for heart failure based on the combined changes in weight and waist circumference using fine‐gray model.
**Table S1:** Associations between weight change categories and heart failure among never smokers (*n* = 31329).
**Table S2:** Associations between waist circumference change and heart failure among never smokers (*n* = 31329).
**Table S3:** Associations between weight change categories and heart failure after excluding 122 heart failure cases within one years (*n* = 45498).
**Table S4:** Associations between waist circumference change and heart failure after excluding 122 heart failure cases within one years (*n* = 45498).
**Table S5:** Associations between weight change categories and heart failure using fine‐gray model.
**Table S6:** Associations between waist circumference change categories and heart failure using fine‐gray model STROBE statement—checklist of items that should be included in reports of cohort studies.
