# Supplementary material for: Changes in Weight, Waist Circumference or Both With Incident Heart Failure in Chinese Middle‐Aged and Older Adults
Source: J Cachexia Sarcopenia Muscle. 2025 Sep 18;16(5):e70059. doi: 10.1002/jcsm.70059 (PMC12445124; doi:10.1002/jcsm.70059)
Supplement: Supplementary file 1 — Figure S1: Flow chart of participant recruitment from the Kailuan study. Figure S2: Cumulative incidence of heart failure by weight change and waist circumference change. Figure S3: Associations between weight change categories and heart failure, stratified by age, sex, bmi, waist circumference, physical activity and dietary pattern. Figure S4: Associations between waist circumference change categories and heart failure, stratified by age, sex, bmi, waist circumference, physical activity and dietary pattern. Figure S5: Adjusted hazard ratios for heart failure stratified by age based on the combined changes in weight and waist circumference. Figure S6: Adjusted hazard ratios for heart failure stratified by sex based on the combined changes in weight and waist circumference. Figure S7: Adjusted hazard ratios for heart failure stratified by BMI based on the combined changes in weight and waist circumference. Figure S8: Adjusted hazard ratios for heart failure stratified by waist circumference based on the combined changes in weight and waist circumference. Figure S9: Adjusted hazard ratios for heart failure stratified by physical activity level based on the combined changes in weight and waist circumference. Figure S10: Adjusted hazard ratios for heart failure stratified by dietary pattern based on the combined changes in weight and waist circumference. Figure S11: Adjusted hazard ratios for heart failure among never smokers based on the combined changes in weight and waist circumference (n = 31329). Figure S12: Adjusted hazard ratios for heart failure based on the combined changes in weight and waist circumference (excluding 122 heart failure cases within one year, n = 45498). Figure S13: Adjusted hazard ratios for heart failure based on the combined changes in weight and waist circumference using fine‐gray model. Table S1: Associations between weight change categories and heart failure among never smokers (n = 31329). Table S2: Associations between waist circumferenc [file JCSM-16-e70059-s001.docx]

Supplemental Online Content

[eFigure 1. Flow Chart of Participant Recruitment from the Kailuan Study. 2](#_Toc199508011)

[eFigure 2. Cumulative incidence of Heart Failure by Weight Change and Waist Circumference Change. 3](#_Toc199508012)

[eFigure 3. Associations Between Weight Change Categories and Heart Failure, Stratified by Age, Sex, BMI, Waist Circumference, Physical Activity, and Dietary Pattern. 4](#_Toc199508013)

[eFigure 4. Associations Between Waist Circumference Change Categories and Heart Failure, Stratified by Age, Sex, BMI, Waist Circumference, Physical Activity, and Dietary Pattern. 6](#_Toc199508014)

[eFigure 5. Adjusted Hazard Ratios for Heart Failure Stratified by Age Based on the Combined Changes in Weight and Waist Circumference. 8](#_Toc199508015)

[eFigure 6. Adjusted Hazard Ratios for Heart Failure Stratified by Sex Based on the Combined Changes in Weight and Waist Circumference. 9](#_Toc199508016)

[eFigure 7. Adjusted Hazard Ratios for Heart Failure Stratified by BMI Based on the Combined Changes in Weight and Waist Circumference. 10](#_Toc199508017)

[eFigure 8. Adjusted Hazard Ratios for Heart Failure Stratified by Waist Circumference Based on the Combined Changes in Weight and Waist Circumference. 11](#_Toc199508018)

[eFigure 9. Adjusted Hazard Ratios for Heart Failure Stratified by Physical Activity Level Based on the Combined Changes in Weight and Waist Circumference. 12](#_Toc199508019)

[eFigure 10. Adjusted Hazard Ratios for Heart Failure Stratified by Dietary Pattern Based on the Combined Changes in Weight and Waist Circumference. 13](#_Toc199508020)

[eFigure 11. Adjusted Hazard Ratios for Heart Failure Among Never Smokers Based on the Combined Changes in Weight and Waist Circumference (n = 31329). 14](#_Toc199508021)

[eFigure 12. Adjusted Hazard Ratios for Heart Failure Based on the Combined Changes in Weight and Waist Circumference (Excluding 122 Heart Failure Cases Within One Year, n = 45498). 15](#_Toc199508022)

[▪ eFigure 13. Adjusted Hazard Ratios for Heart Failure Based on the Combined Changes in Weight and Waist Circumference using Fine-Gray model. 16](#_Toc199508023)

[▪eTable 1. Associations Between Weight Change Categories and Heart Failure Among Never Smokers (n = 31329). 17](#_Toc199508024)

[▪eTable 2. Associations Between Waist Circumference Change and Heart Failure Among Never Smokers (n = 31329). 17](#_Toc199508025)

[▪eTable 3. Associations Between Weight Change Categories and Heart Failure after Excluding 122 Heart Failure Cases Within One Years (n = 45498). 18](#_Toc199508026)

[▪eTable 4. Associations Between Waist Circumference Change and Heart Failure after Excluding 122 Heart Failure Cases Within One Years (n = 45498). 18](#_Toc199508027)

[▪eTable 5. Associations between Weight Change Categories and Heart Failure using Fine-Gray model. 19](#_Toc199508028)

[▪eTable 6. Associations between Waist Circumference Change Categories and Heart Failure using Fine-Gray model. 20](#_Toc199508029)

[STROBE Statement—Checklist of items that should be included in reports of *cohort studies* 21](#_Toc199508030)

eFigure 1. Flow Chart of Participant Recruitment from the Kailuan Study.

**
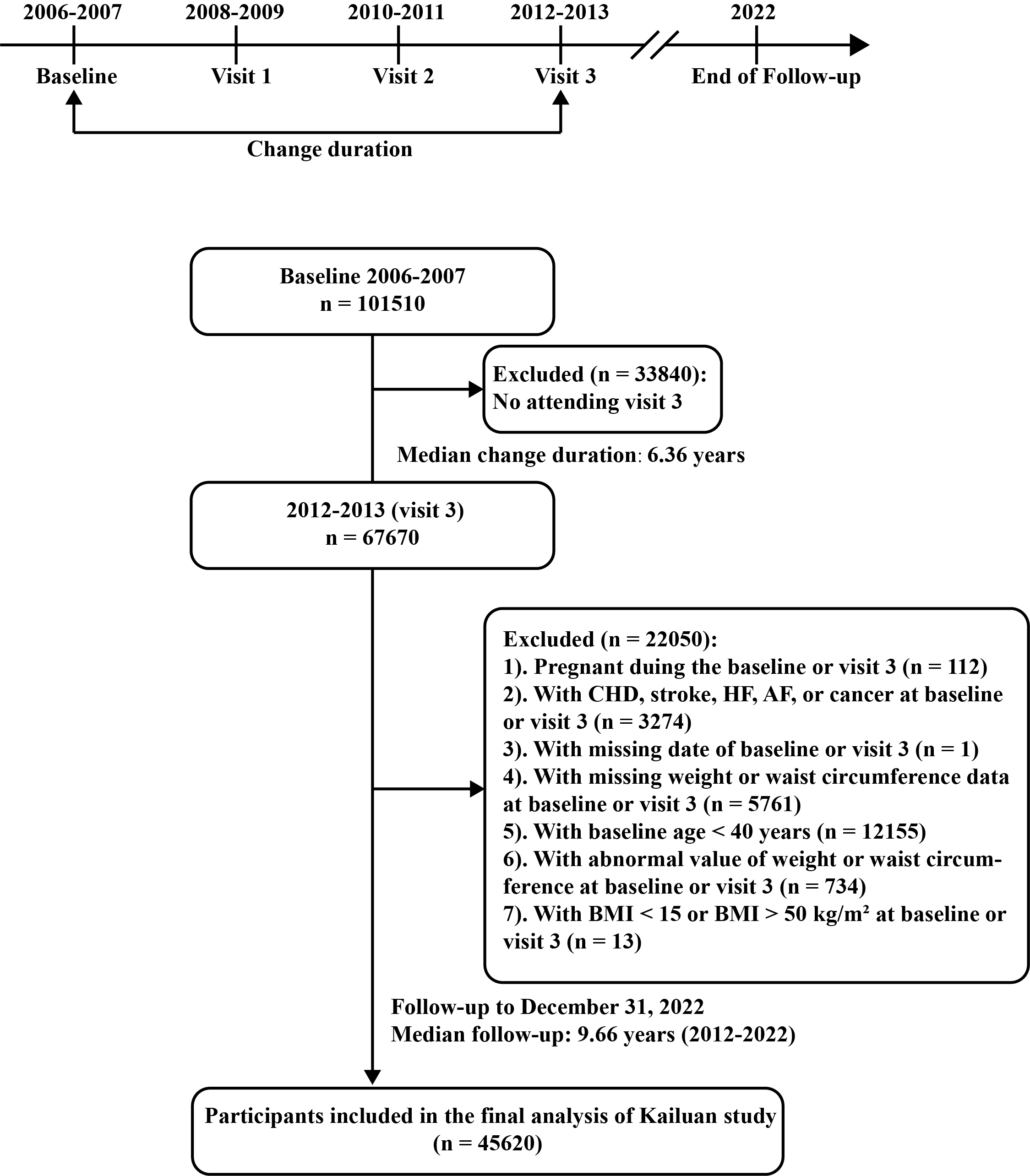
**

# eFigure 2. Cumulative incidence of Heart Failure by Weight Change and Waist Circumference Change.


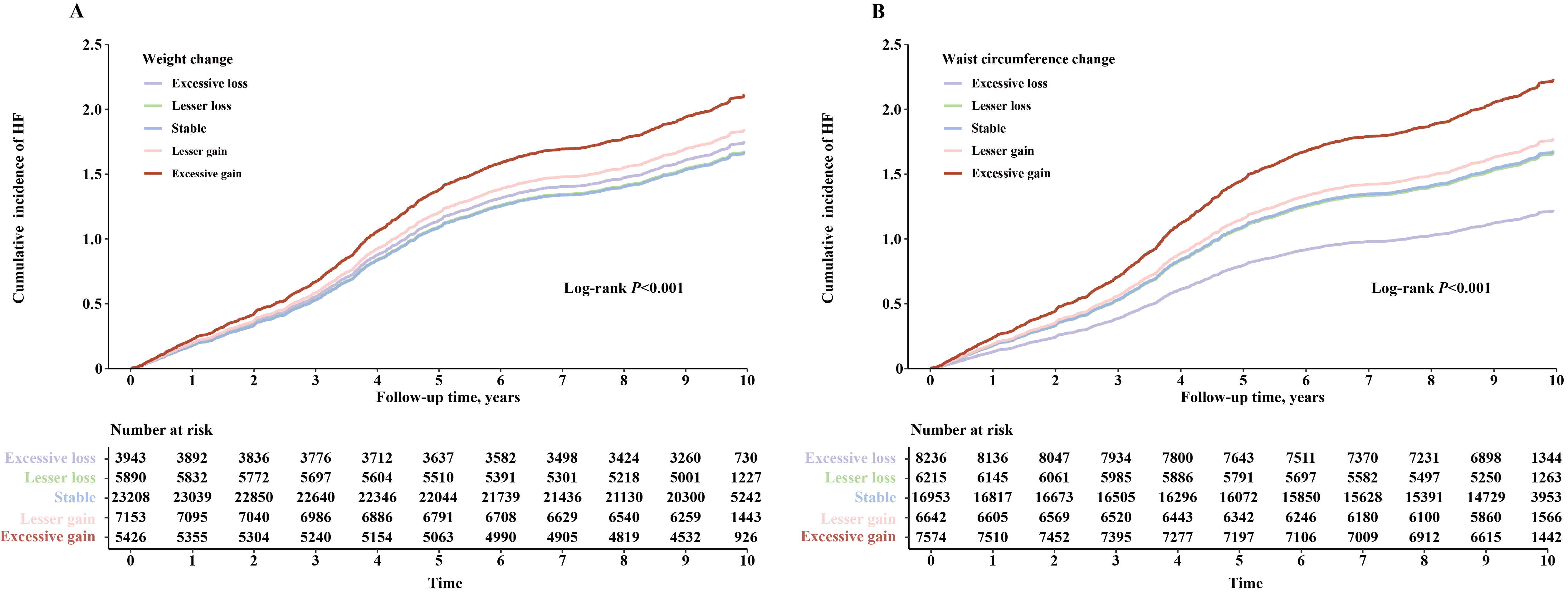


**Figure Legend**. The figure shows the adjusted Kaplan–Meier cumulative incidence curves (adjusted for age, sex, height, weight at cohort recruitment, and waist circumference change for weight change. Adjusted for age, sex, height, waist circumference at cohort recruitment, and weight change for waist circumference change) for heart failure according to weight change (A), waist circumference change (B).

#
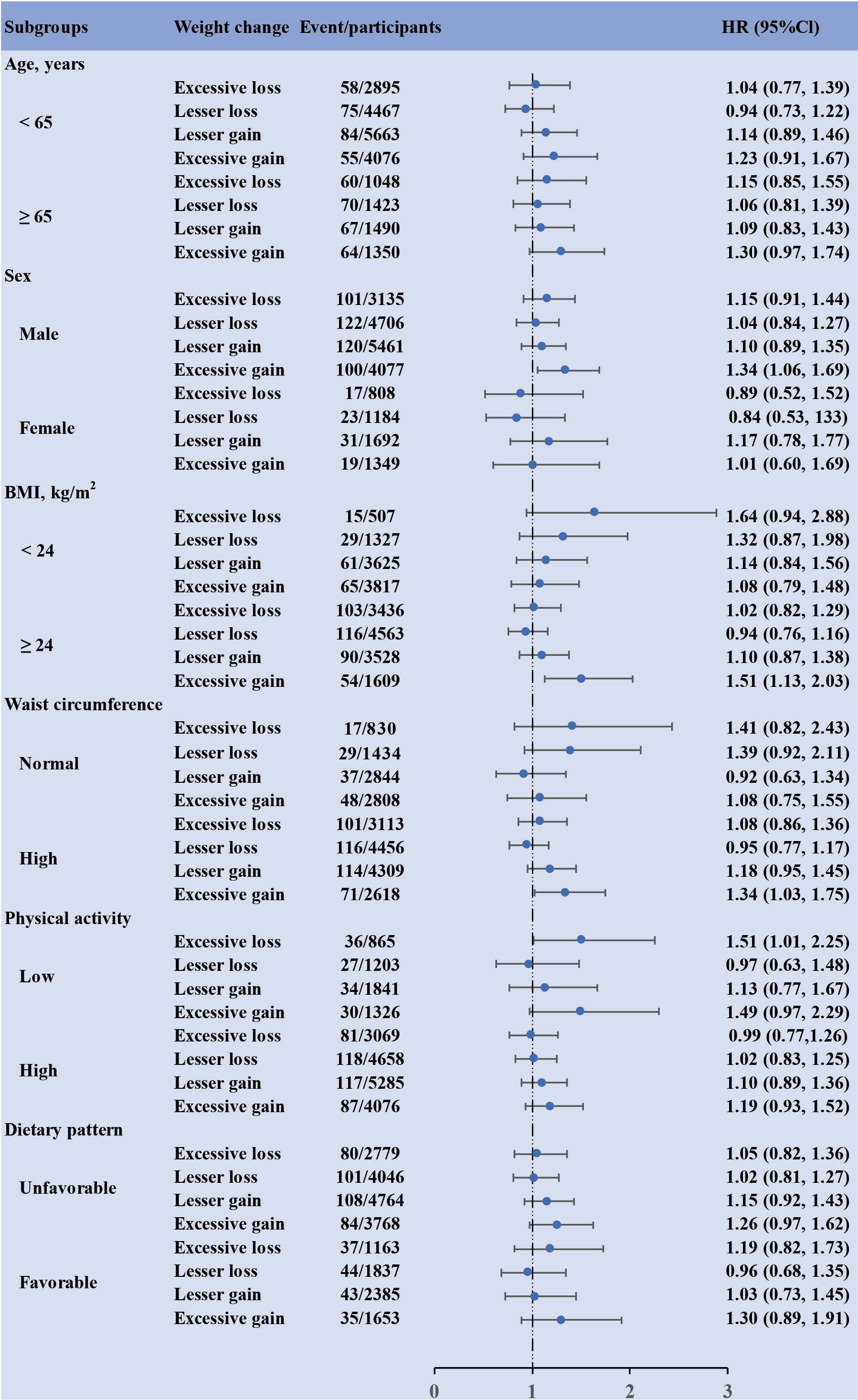
eFigure 3. Associations Between Weight Change Categories and Heart Failure, Stratified by Age, Sex, BMI, Waist Circumference, Physical Activity, and Dietary Pattern.

**Figure Legend**. The multivariable models were adjusted for height and weight at cohort recruitment, waist circumference change (continuous variables), smoking status, alcohol intake status, dietary pattern, educational attainment, physical activity, occupation, hypertension, fasting blood glucose, total serum cholesterol level, and stratified by age at risk (5-year interval) and sex. Each group was adjusted for other covariates except itself. Weight changes were categorized into five groups: Excessive weight loss (lost > 10%), lesser weight loss (10% ≤ lost < 5%), stable weight (change within 5%), lesser weight gain (5% < gained ≤ 10%), and excessive weight gain (gained > 10%). Normal waist circumferences were defined as the waist circumference of males < 85 cm or females < 80 cm. High waist circumferences were defined as the waist circumference of males ≥ 85 cm or females ≥ 80 cm. In the Kailuan study, low physical activity was defined as no physical activity, and high physical activity was defined as occasional and regular physical activity. Unfavorable pattern was defined as unfavorable pattern, and favorable pattern was defined as favorable pattern and intermediate pattern.

**
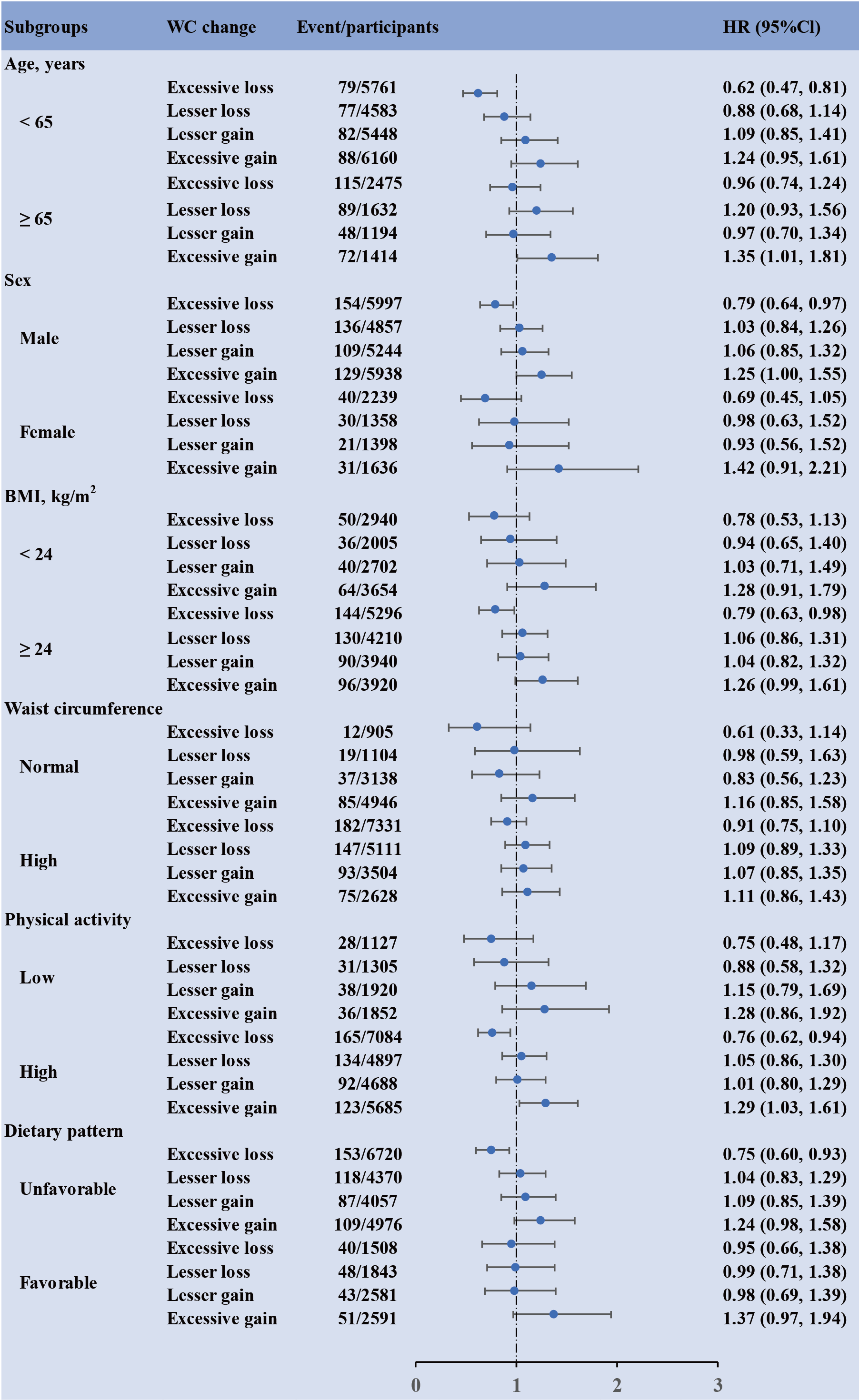
eFigure 4. Associations Between Waist Circumference Change Categories and Heart Failure, Stratified by Age, Sex, BMI, Waist Circumference, Physical Activity, and Dietary Pattern.**

**Figure Legend**. The multivariable models were adjusted for height and waist circumference at cohort recruitment, weight circumference change (continuous variables), smoking status, alcohol intake status, dietary pattern, educational attainment, physical activity, occupation, hypertension, fasting blood glucose, total serum cholesterol level, and stratified by age at risk (5-year interval) and sex. Each group was adjusted for other covariates except itself. Waist circumference changes were categorized into five groups: Excessive waist circumference loss (lost > 10%), lesser waist circumference loss (10% ≤ lost < 5%), stable waist circumference (change within 5%), lesser waist circumference gain (5% < gained ≤ 10%), and excessive waist circumference gain (gained > 10%). Normal waist circumferences were defined as the waist circumference of males < 85 cm or females < 80 cm. High waist circumferences were defined as the waist circumference of males ≥ 85 cm or females ≥ 80cm. In the Kailuan study, low physical activity was defined as no physical activity, and high physical activity was defined as occasional and regular physical activity. Unfavorable pattern was defined as unfavorable pattern, and favorable pattern was defined as favorable pattern and intermediate pattern.

**

eFigure 5. Adjusted Hazard Ratios for Heart Failure Stratified by Age Based on the Combined Changes in Weight and Waist Circumference.**

**Figure Legend.** The multivariable-adjusted model included the combined categories of weight and waist circumference changes, weight, height, and waist circumference at cohort recruitment, smoking status, alcohol intake status, dietary pattern, educational attainment, physical activity, occupation, hypertension, fasting blood glucose, total serum cholesterol level; and stratified by age at risk (5-year interval) and sex. Weight changes were categorized into five groups: Excessive weight loss (lost > 10%), lesser weight loss (10% ≤ lost < 5%), stable weight (change within 5%), lesser weight gain (5% < gained ≤ 10%), and excessive weight gain (gained > 10%). Waist circumference changes were categorized into five groups: Excessive waist circumference loss (lost > 10%), lesser waist circumference loss (10% ≤ lost < 5%), stable waist circumference (change within 5%), lesser waist circumference gain (5% < gained ≤ 10%), and excessive waist circumference gain (gained > 10%).

**

eFigure 6. Adjusted Hazard Ratios for Heart Failure Stratified by Sex Based on the Combined Changes in Weight and Waist Circumference.**

**Figure Legend.** The multivariable-adjusted model included the combined categories of weight and waist circumference changes, weight, height, and waist circumference at cohort recruitment, smoking status, alcohol intake status, dietary pattern, educational attainment, physical activity, occupation, hypertension, fasting blood glucose, total serum cholesterol level; and stratified by age at risk (5-year interval). Weight changes were categorized into five groups: Excessive weight loss (lost > 10%), lesser weight loss (10% ≤ lost < 5%), stable weight (change within 5%), lesser weight gain (5% < gained ≤ 10%), and excessive weight gain (gained > 10%). Waist circumference changes were categorized into five groups: Excessive waist circumference loss (lost > 10%), lesser waist circumference loss (10% ≤ lost < 5%), stable waist circumference (change within 5%), lesser waist circumference gain (5% < gained ≤ 10%), and excessive waist circumference gain (gained > 10%).

**

eFigure 7. Adjusted Hazard Ratios for Heart Failure Stratified by BMI Based on the Combined Changes in Weight and Waist Circumference.**

**Figure Legend.** The multivariable-adjusted model included the combined categories of weight and waist circumference changes, weight, height, and waist circumference at cohort recruitment, smoking status, alcohol intake status, dietary pattern, educational attainment, physical activity, occupation, hypertension, fasting blood glucose, total serum cholesterol level; and stratified by age at risk (5-year interval) and sex. BMI was categorized into < 24 kg/m^2^ and ≥ 24 kg/m^2^. Weight changes were categorized into five groups: Excessive weight loss (lost > 10%), lesser weight loss (10% ≤ lost < 5%), stable weight (change within 5%), lesser weight gain (5% < gained ≤ 10%), and excessive weight gain (gained > 10%). Waist circumference changes were categorized into five groups: Excessive waist circumference loss (lost > 10%), lesser waist circumference loss (10% ≤ lost < 5%), stable waist circumference (change within 5%), lesser waist circumference gain (5% < gained ≤ 10%), and excessive waist circumference gain (gained > 10%).

**

eFigure 8. Adjusted Hazard Ratios for Heart Failure Stratified by Waist Circumference Based on the Combined Changes in Weight and Waist Circumference.**

**Figure Legend.** The multivariable-adjusted model included the combined categories of weight and waist circumference changes, weight, height at cohort recruitment, smoking status, alcohol intake status, dietary pattern, educational attainment, physical activity, occupation, hypertension, fasting blood glucose, total serum cholesterol level; and stratified by age at risk (5-year interval) and sex. Normal waist circumferences were defined as the waist circumference of male < 85 cm or female < 80 cm. High waist circumferences were defined as the waist circumference of male ≥ 85 cm or female ≥ 80 cm. Weight changes were categorized into five groups: Excessive weight loss (lost > 10%), lesser weight loss (10% ≤ lost < 5%), stable weight (change within 5%), lesser weight gain (5% < gained ≤ 10%), and excessive weight gain (gained > 10%). Waist circumference changes were categorized into five groups: Excessive waist circumference loss (lost > 10%), lesser waist circumference loss (10% ≤ lost < 5%), stable waist circumference (change within 5%), lesser waist circumference gain (5% < gained ≤ 10%), and excessive waist circumference gain (gained > 10%).

**

eFigure 9. Adjusted Hazard Ratios for Heart Failure Stratified by Physical Activity Level Based on the Combined Changes in Weight and Waist Circumference.**

**Figure Legend.** The multivariable-adjusted model included the combined categories of weight and waist circumference changes, weight, height and waist circumference at cohort recruitment, smoking status, alcohol intake status, dietary pattern, educational attainment, occupation, hypertension, fasting blood glucose, total serum cholesterol level; and stratified by age at risk (5-year interval) and sex. In the Kailuan study, low physical activity was defined as no physical activity, and high physical activity was defined as occasional and regular physical activity. Weight changes were categorized into five groups: Excessive weight loss (lost > 10%), lesser weight loss (10% ≤ lost < 5%), stable weight (change within 5%), lesser weight gain (5% < gained ≤ 10%), and excessive weight gain (gained > 10%). Waist circumference changes were categorized into five groups: Excessive waist circumference loss (lost > 10%), lesser waist circumference loss (10% ≤ lost < 5%), stable waist circumference (change within 5%), lesser waist circumference gain (5% < gained ≤ 10%), and excessive waist circumference gain (gained > 10%).

**

eFigure 10. Adjusted Hazard Ratios for Heart Failure Stratified by Dietary Pattern Based on the Combined Changes in Weight and Waist Circumference.**

**Figure Legend.** The multivariable-adjusted model included the combined categories of weight and waist circumference changes, weight, height, and waist circumference at cohort recruitment, smoking status, alcohol intake status, educational attainment, physical activity, occupation, hypertension, fasting blood glucose, total serum cholesterol level; and stratified by age at risk (5-year interval) and sex. Unfavorable pattern was defined as unfavorable pattern, and favorable pattern was defined as favorable pattern and intermediate pattern. Weight changes were categorized into five groups: Excessive weight loss (lost > 10%), lesser weight loss (10% ≤ lost < 5%), stable weight (change within 5%), lesser weight gain (5% < gained ≤ 10%), and excessive weight gain (gained > 10%). Waist circumference changes were categorized into five groups: Excessive waist circumference loss (lost > 10%), lesser waist circumference loss (10% ≤ lost < 5%), stable waist circumference (change within 5%), lesser waist circumference gain (5% < gained ≤ 10%), and excessive waist circumference gain (gained > 10%).

**eFigure 11. Adjusted Hazard Ratios for Heart Failure Among Never Smokers Based on the Combined Changes in Weight and Waist Circumference (n = 31329).
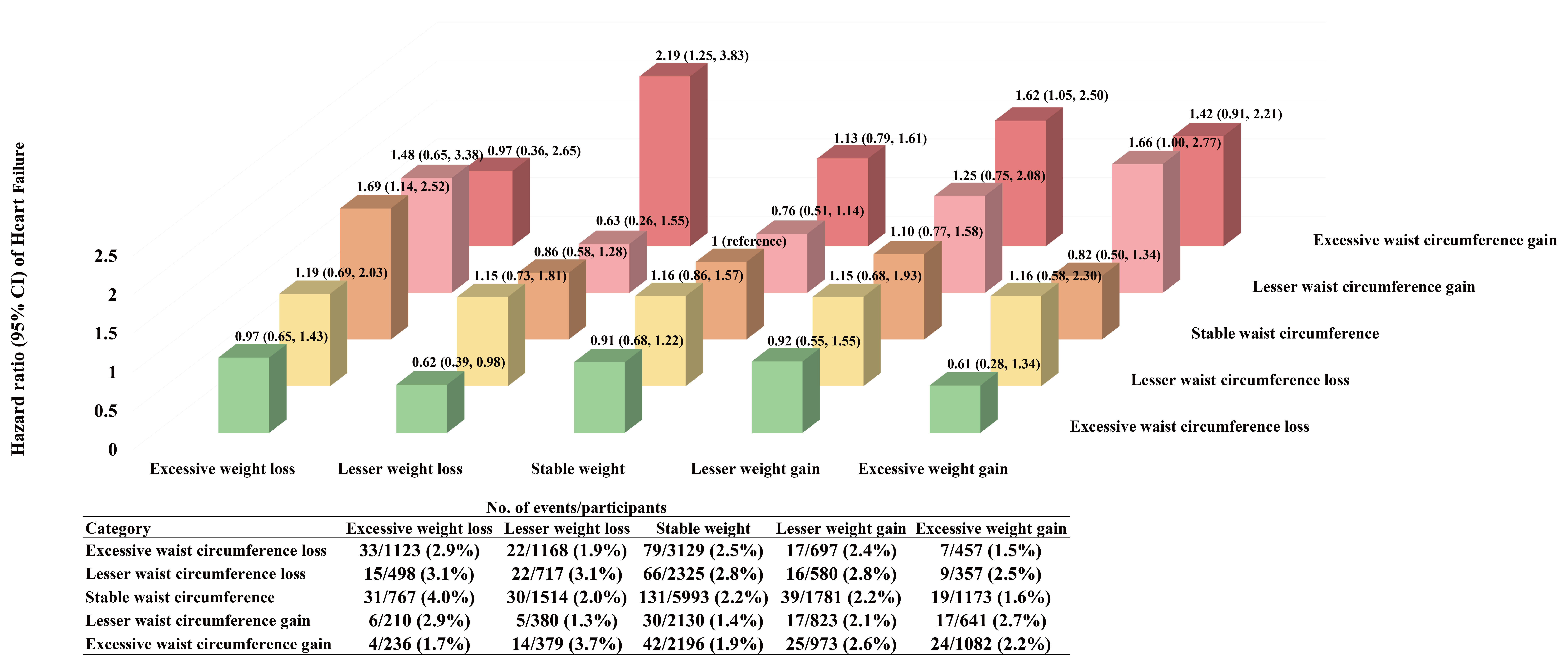
**

**Figure Legend.** The multivariable-adjusted model included the combined categories of weight and waist circumference changes, weight, height, and waist circumference at cohort recruitment, alcohol intake status, dietary pattern, educational attainment, physical activity, occupation, hypertension, fasting blood glucose, total serum cholesterol level; and stratified by age at risk (5-year interval) and sex. Weight changes were categorized into five groups: Excessive weight loss (lost > 10%), lesser weight loss (10% ≤ lost < 5%), stable weight (change within 5%), lesser weight gain (5% < gained ≤ 10%), and excessive weight gain (gained > 10%). Waist circumference changes were categorized into five groups: Excessive waist circumference loss (lost > 10%), lesser waist circumference loss (10% ≤ lost < 5%), stable waist circumference (change within 5%), lesser waist circumference gain (5% < gained ≤ 10%), and excessive waist circumference gain (gained > 10%).

**
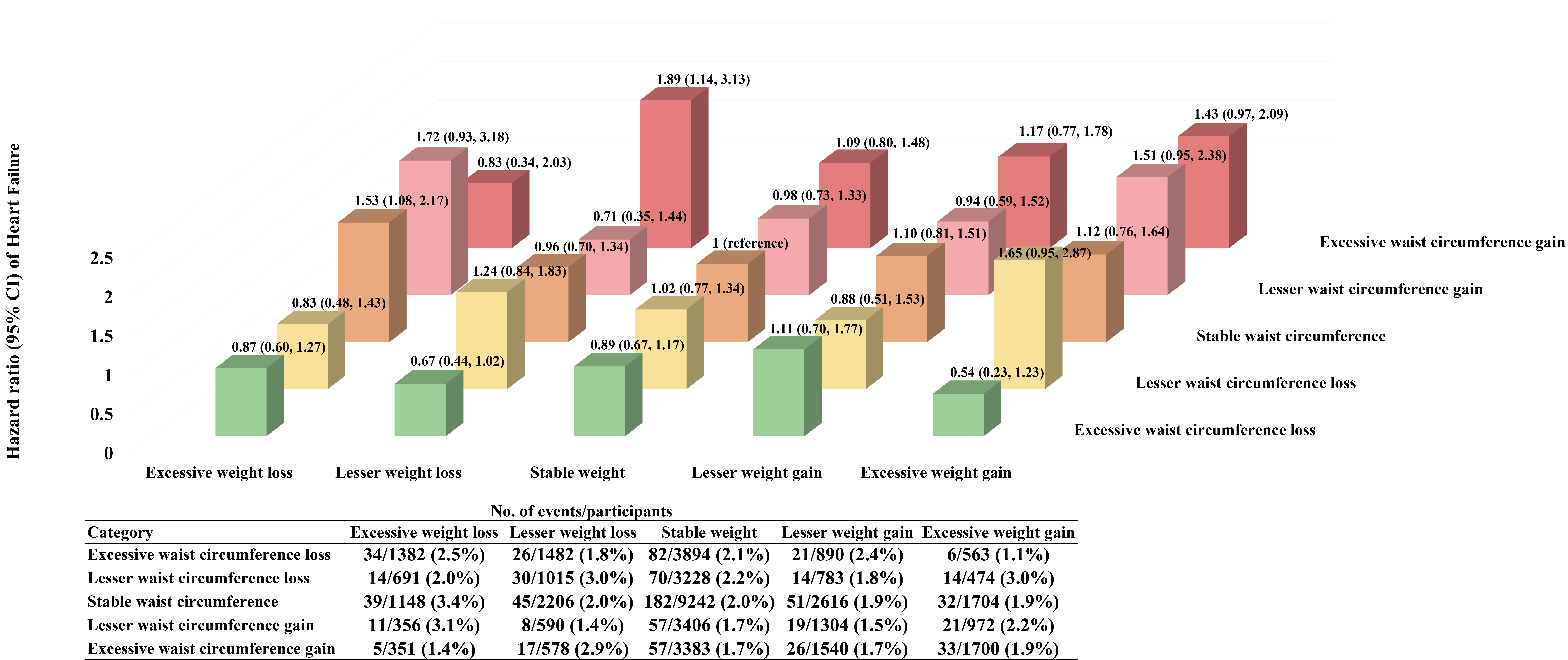
eFigure 12. Adjusted Hazard Ratios for Heart Failure Based on the Combined Changes in Weight and Waist Circumference (Excluding 122 Heart Failure Cases Within One Year, n = 45498).**

**Figure Legend.** The multivariable-adjusted model included the combined categories of weight and waist circumference changes, weight, height, and waist circumference at cohort recruitment, smoking status, alcohol intake status, dietary pattern, educational attainment, physical activity, occupation, hypertension, fasting blood glucose, total serum cholesterol level; and stratified by age at risk (5-year interval) and sex. Weight changes were categorized into five groups: Excessive weight loss (lost > 10%), lesser weight loss (10% ≤ lost < 5%), stable weight (change within 5%), lesser weight gain (5% < gained ≤ 10%), and excessive weight gain (gained > 10%). Waist circumference changes were categorized into five groups: Excessive waist circumference loss (lost > 10%), lesser waist circumference loss (10% ≤ lost < 5%), stable waist circumference (change within 5%), lesser waist circumference gain (5% < gained ≤ 10%), and excessive waist circumference gain (gained > 10%).

**▪ eFigure 13. Adjusted Hazard Ratios for Heart Failure Based on the Combined Changes in Weight and Waist Circumference** **using Fine-Gray model.**

**
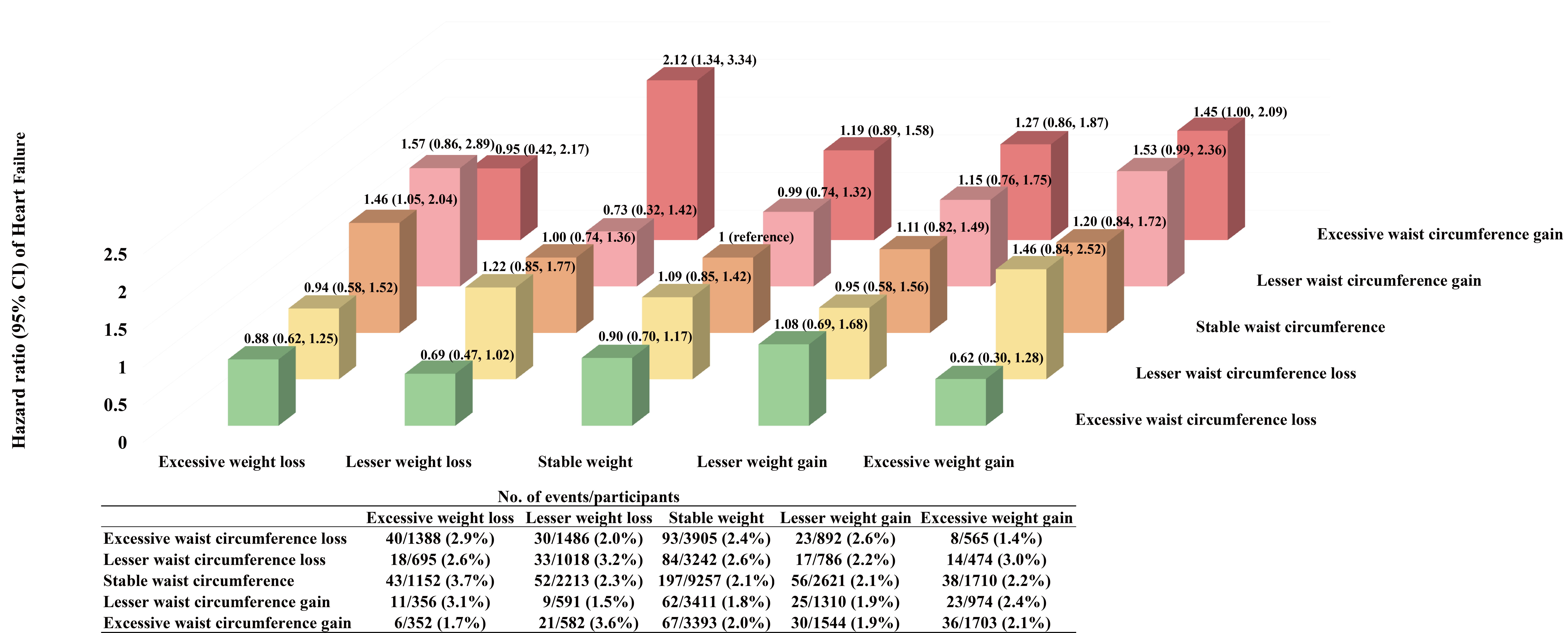
**

**Figure Legend.** The multivariable-adjusted model included the combined categories of weight and waist circumference changes, weight, height, and waist circumference at cohort recruitment, smoking status, alcohol intake status, dietary pattern, educational attainment, physical activity, occupation, hypertension, fasting blood glucose, total serum cholesterol level, age, and sex. Weight changes were categorized into five groups: Excessive weight loss (lost > 10%), lesser weight loss (10% ≤ lost < 5%), stable weight (change within 5%), lesser weight gain (5% < gained ≤ 10%), and excessive weight gain (gained > 10%). Waist circumference changes were categorized into five groups: Excessive waist circumference loss (lost > 10%), lesser waist circumference loss (10% ≤ lost < 5%), stable waist circumference (change within 5%), lesser waist circumference gain (5% < gained ≤ 10%), and excessive waist circumference gain (gained > 10%).

**▪eTable 1. Associations Between Weight Change Categories and Heart Failure Among Never Smokers (n = 31329).**

|  | Weight change (%) | | | | |
| --- | --- | --- | --- | --- | --- |
| Variable | Loss | | Stable | Gain | |
|  | (change < -10) | (-10 ≤ change < -5) | (-5 ≤ change ≤ 5) | (5 < change ≤ 10) | (change > 10) |
| No. of events/total | 89/2834 | 93/4158 | 348/15773 | 114/4854 | 76/3710 |
| Incidence rate, per 1000 person-years | 3.52 (2.86, 4.33) | 2.47 (2.01, 3.02) | 2.40 (2.16, 2.66) | 2.55 (2.13, 3.07) | 2.27 (1.81, 2.84) |
| HR (95% CI) | 1.22 (0.95, 1.56) | 0.91 (0.72, 1.15) | 1 (reference) | 1.19 (0.96, 1.47) | 1.10 (0.85, 1.43) |

The multivariable models were adjusted for height and weight at cohort recruitment, waist circumference change (continuous variables), alcohol intake status, dietary pattern, educational attainment, physical activity, occupation, hypertension, fasting blood glucose, total serum cholesterol level, and stratified by age at risk (5-year interval) and sex.

Abbreviations: HR, hazard ratio; CI, confidence interval.

**▪eTable 2. Associations Between Waist Circumference Change and Heart Failure Among Never Smokers (n = 31329).**

|  | Waist Circumference change (%) | | | | |
| --- | --- | --- | --- | --- | --- |
| Variable | Loss | | Stable | Gain | |
|  | (change < -10) | (-10 ≤ change < -5) | (-5 ≤ change ≤ 5) | (5 < change ≤ 10) | (change > 10) |
| No. of events/total | 158/6574 | 128/4477 | 250/11228 | 75/4184 | 109/4866 |
| Incidence rate, per 1000 person-years | 2.67 (2.29, 3.12) | 3.17 (2.66, 3.77) | 2.42 (2.14, 2.74) | 1.93 (1.54, 2.42) | 2.44 (2.02, 2.94) |
| HR (95% CI) | **0.80 (0.65, 0.99)** | 1.12 (0.90, 1.39) | 1 (reference) | 0.98 (0.76, 1.27) | **1.35 (1.06, 1.71)** |

The multivariable models were adjusted for height and waist circumference at cohort recruitment, weight change (continuous variables), alcohol intake status, dietary pattern, educational attainment, physical activity, occupation, hypertension, fasting blood glucose, total serum cholesterol level, and stratified by age at risk (5-year interval) and sex.

Abbreviations: HR, hazard ratio; CI, confidence interval.

**▪eTable 3. Associations Between Weight Change Categories and Heart Failure after Excluding 122 Heart Failure Cases Within One Years (n = 45498).**

|  | Weight change (%) | | | | |
| --- | --- | --- | --- | --- | --- |
| Variable | Loss | | Stable | Gain | |
|  | (change < -10) | (-10 ≤ change < -5) | (-5 ≤ change ≤ 5) | (5 < change ≤ 10) | (change > 10) |
| No. of events/total | 103/3928 | 126/5871 | 448/23153 | 131/7133 | 106/5413 |
| Incidence rate, per 1000 person-years | 2.91 (2.40, 3.53) | 2.35 (1.97, 2.80) | 2.08 (1.90, 2.29) | 1.98 (1.67, 2.35) | 2.16 (1.78, 2.61) |
| HR (95% CI) | 1.10 (0.88, 1.38) | 0.99 (0.81, 1.21) | 1 (reference) | 1.18 (0.88, 1.31) | **1.25 (1.00, 1.57)** |

The multivariable models were adjusted for height and weight at cohort recruitment, waist circumference change (continuous variables), smoking status, alcohol intake status, dietary pattern, educational attainment, physical activity, occupation, hypertension, fasting blood glucose, total serum cholesterol level, and stratified by age at risk (5-year interval) and sex.

Abbreviations: HR, hazard ratio; CI, confidence interval.

**▪eTable 4. Associations Between Waist Circumference Change and Heart Failure after Excluding 122 Heart Failure Cases Within One Years (n = 45498).**

|  | Waist Circumference change (%) | | | | |
| --- | --- | --- | --- | --- | --- |
| Variable | Loss | | Stable | Gain | |
|  | (change < -10) | (-10 ≤ change < -5) | (-5 ≤ change ≤ 5) | (5 < change ≤ 10) | (change > 10) |
| No. of events/total | 169/8211 | 142/6191 | 349/16916 | 116/6628 | 138/7552 |
| Incidence rate, per 1000 person-years | 2.27 (1.95, 2.64) | 2.52 (2.13, 2.97) | 2.23 (2.01, 2.47) | 1.87 (1.56, 2.25) | 1.97 (1.67, 2.33) |
| HR (95% CI) | **0.76 (0.62, 0.92)** | 0.98 (0.80, 1.19) | 1 (reference) | 1.02 (0.82, 1.26) | 1.19 (0.97, 1.47) |

The multivariable models were adjusted for height and waist circumference at cohort recruitment, weight change (continuous variables), smoking status, alcohol intake status, dietary pattern, educational attainment, physical activity, occupation, hypertension, fasting blood glucose, total serum cholesterol level, and stratified by age at risk (5-year interval) and sex.

Abbreviations: HR, hazard ratio; CI, confidence interval.

**▪eTable 5. Associations between Weight Change Categories and Heart Failure using Fine-Gray model.**

|  | Weight change (%) | | | | |
| --- | --- | --- | --- | --- | --- |
| Variable | Loss | | Stable | Gain | |
|  | (change < -10) | (-10 ≤ change < -5) | (-5 ≤ change ≤ 5) | (5 < change ≤ 10) | (change > 10) |
| No. of events/total | 118/3943 | 145/5890 | 503/23208 | 151/7153 | 119/5426 |
| Incidence rate, per 1000 person-years | 3.33 (2.78, 3.99) | 2.70 (2.30, 3.18) | 2.34 (2.14, 2.55) | 2.28 (1.95, 2.68) | 2.42 (2.02, 2.90) |
| HR (95% CI) |  |  |  |  |  |
| Model 1 | 1.03 (0.83, 1.28) | 1.00 (0.83 1.21) | 1 (reference) | 1.11 (0.92, 1.33) | **1.25 (1.02, 1.55)** |
| Model 2 | 1.01 (0.81, 1.25) | 0.99 (0.82, 1.20) | 1 (reference) | 1.11 (0.92, 1.33) | **1.26 (1.02, 1.55)** |
| Model 3 | 1.07 (0.87, 1.33) | 0.99 (0.82, 1.20) | 1 (reference) | 1.10 (0.92, 1.32) | **1.25 (1.01, 1.54)** |

Model 1: The multivariable models were adjusted for height and weight at cohort recruitment, waist circumference change (continuous variables), age, and sex.

Model 2: The multivariable models were adjusted for height and weight at cohort recruitment, waist circumference change (continuous variables), smoking status, alcohol intake status, dietary pattern, educational attainment, physical activity, age, and sex.

Model 3: The multivariable models were adjusted for height and weight at cohort recruitment, waist circumference change (continuous variables), smoking status, alcohol intake status, dietary pattern, educational attainment, physical activity, occupation, hypertension, fasting blood glucose, total serum cholesterol level, age, and sex.

Abbreviations: HR, hazard ratio; CI, confidence interval.

**▪eTable 6. Associations between Waist Circumference Change Categories and Heart Failure using Fine-Gray model.**

|  | Waist Circumference change (%) | | | | |
| --- | --- | --- | --- | --- | --- |
| Variable | Loss | | Stable | Gain | |
|  | (change < -10) | (-10 ≤ change < -5) | (-5 ≤ change ≤ 5) | (5 < change ≤ 10) | (change > 10) |
| No. of events/total | 194/8236 | 166/6215 | 386/16953 | 130/6642 | 160/7574 |
| Incidence rate, per 1000 person-years | 2.61 (2.26, 3.00) | 2.94 (2.53, 3.42) | 2.46 (2.23, 2.72) | 2.10 (1.77, 2.49) | 2.29 (1.96, 2.67) |
| HR (95% CI) |  |  |  |  |  |
| Model 1 | **0.72 (0.60, 0.86)** | 0.99 (0.82, 1.18) | 1 (reference) | 1.05 (0.86, 1.29) | **1.33 (1.09, 1.61)** |
| Model 2 | **0.71 (0.59, 0.86)** | 0.99 (0.82, 1.19) | 1 (reference) | 1.05 (0.86, 1.29) | **1.32 (1.09, 1.61)** |
| Model 3 | **0.76 (0.63, 0.91)** | 1.01 (0.84, 1.21) | 1 (reference) | 1.04 (0.85, 1.28) | **1.28 (1.05, 1.55)** |

Model 1: The multivariable models were adjusted for height and waist circumference at cohort recruitment, weight change (continuous variables), age, and sex.

Model 2: The multivariable models were adjusted for height and waist circumference at cohort recruitment, weight change (continuous variables), smoking status, alcohol intake status, dietary pattern, educational attainment, physical activity, age, and sex.

Model 3: The multivariable models were adjusted for height and waist circumference at cohort recruitment, weight change (continuous variables), smoking status, alcohol intake status, dietary pattern, educational attainment, physical activity, occupation, hypertension, fasting blood glucose, total serum cholesterol level, age, and sex.

Abbreviations: HR, hazard ratio; CI, confidence interval.

STROBE Statement—Checklist of items that should be included in reports of *cohort studies*

|  | Item No | Recommendation | Page No |
| --- | --- | --- | --- |
| **Title and abstract** | 1 | (*a*) Indicate the study’s design with a commonly used term in the title or the abstract | Page 2 |
|  |  | (*b*) Provide in the abstract an informative and balanced summary of what was done and what was found | Page 2 to 4 |
| Introduction | | | |
| Background/rationale | 2 | Explain the scientific background and rationale for the investigation being reported | Page 5 to 6 |
| Objectives | 3 | State specific objectives, including any prespecified hypotheses | Page 6 |
| Methods | | | |
| Study design | 4 | Present key elements of study design early in the paper | Page 6 |
| Setting | 5 | Describe the setting, locations, and relevant dates, including periods of recruitment, exposure, follow-up, and data collection | Page 6 to 10 |
| Participants | 6 | (*a*) Give the eligibility criteria, and the sources and methods of selection of participants. Describe methods of follow-up | Page 7 to 8 |
|  |  | (*b*) For matched studies, give matching criteria and number of exposed and unexposed | NA |
| Variables | 7 | Clearly define all outcomes, exposures, predictors, potential confounders, and effect modifiers. Give diagnostic criteria, if applicable | Page 7 to 10, |
| Data sources/ measurement | 8* | For each variable of interest, give sources of data and details of methods of assessment (measurement). Describe comparability of assessment methods if there is more than one group | Page 7 to 10 |
| Bias | 9 | Describe any efforts to address potential sources of bias | Page 8 to 10 |
| Study size | 10 | Explain how the study size was arrived at | Page 6 to 7,  Supplemental  Page 2 |
| Quantitative variables | 11 | Explain how quantitative variables were handled in the analyses. If applicable, describe which groupings were chosen and why | Page 8 to 10 |
| Statistical methods | 12 | (*a*) Describe all statistical methods, including those used to control for confounding | Page 10 to 12 |
|  |  | (*b*) Describe any methods used to examine subgroups and interactions | Page 11 to 12, |
|  |  | (*c*) Explain how missing data were addressed | Page 12 |
|  |  | (*d*) If applicable, explain how loss to follow-up was addressed | NA |
|  |  | (*e*) Describe any sensitivity analyses | Page 12, Supplemental  Material page 17 to 20 |
| Results | | |  |
| Participants | 13* | (a) Report numbers of individuals at each stage of study—eg numbers potentially eligible, examined for eligibility, confirmed eligible, included in the study, completing follow-up, and analysed | Supplemental  Material page 2 (flow chart) |
|  |  | (b) Give reasons for non-participation at each stage | Page 6 to 7 |
|  |  | (c) Consider use of a flow diagram | Supplemental  Material page 2 (flow chart) |
| Descriptive data | 14* | (a) Give characteristics of study participants (eg demographic, clinical, social) and information on exposures and potential confounders | Page 12 to 13 |
|  |  | (b) Indicate number of participants with missing data for each variable of interest | Table 1, Table 2 (page 27-30) |
|  |  | (c) Summarise follow-up time (eg, average and total amount) | Page 12, Supplemental  Material page 2 |
| Outcome data | 15* | Report numbers of outcome events or summary measures over time | Page 12 |

| Main results | 16 | (*a*) Give unadjusted estimates and, if applicable, confounder-adjusted estimates and their precision (eg, 95% confidence interval). Make clear which confounders were adjusted for and why they were included | Page 13 to 14 |
| --- | --- | --- | --- |
|  |  | (*b*) Report category boundaries when continuous variables were categorized | Page 7 |
|  |  | (*c*) If relevant, consider translating estimates of relative risk into absolute risk for a meaningful time period | NA |
| Other analyses | 17 | Report other analyses done—eg analyses of subgroups and interactions, and sensitivity analyses | Page 14 |
| Discussion | | | |
| Key results | 18 | Summarise key results with reference to study objectives | Page 15 |
| Limitations | 19 | Discuss limitations of the study, taking into account sources of potential bias or imprecision. Discuss both direction and magnitude of any potential bias | Page 19 |
| Interpretation | 20 | Give a cautious overall interpretation of results considering objectives, limitations, multiplicity of analyses, results from similar studies, and other relevant evidence | Page 15 to 19 |
| Generalisability | 21 | Discuss the generalisability (external validity) of the study results | Page 19 |
| Other information | | | |
| Funding | 22 | Give the source of funding and the role of the funders for the present study and, if applicable, for the original study on which the present article is based | Page 21 |

*Give information separately for exposed and unexposed groups.

**Note:** An Explanation and Elaboration article discusses each checklist item and gives methodological background and published examples of transparent reporting. The STROBE checklist is best used in conjunction with this article (freely available on the Web sites of PLoS Medicine at http://www.plosmedicine.org/, Annals of Internal Medicine at http://www.annals.org/, and Epidemiology at http://www.epidem.com/). Information on the STROBE Initiative is available at http://www.strobe-statement.org.
